# Inpatient versus outpatient management of young infants with a single low-mortality-risk sign of possible serious bacterial infection in sub-Saharan Africa and south Asia: an open-label, multicentre, two-arm, randomised controlled trial

**DOI:** 10.1016/S2214-109X(25)00243-8

**Published:** 2025-10-15

**Authors:** PSBI Study Group, Abdullah H Baqui, Mohammod Shahidullah, Salahuddin Ahmed, Arunangshu Dutta Roy, Rasheda Khanam, Nabidul Haque Chowdhury, Sabina Ashrafee Lipi, Md Jahurul Islam, Manajjir Ali, Amha Mekasha, Abiy Seifu Estifanos, Lulu Muhe, Damen Hailemariam, Dorka Woldesenbet Keraga, Tabot Keskis Azeze, Bogale Worku, Solome Jebessa, Temsunaro Rongsen-Chandola, Nidhi Goyal, Amit Kumar, Nita Bhandari, Uma Chandra Mouli Natchu, Manisha Gupta, Aritra Guha, Shayam Kaushik, Surjeet Kumar, Amitabh Jain, Mangla Sood, Rakesh Sharma, Jagjit Singh Dalal, Kundan Mittal, GP Kaushal, Vineeta Wadhwa, Anju Seth, Varinder Singh, Harish Pemde, Praveen Kumar, Viswas Chhapola, Yashwant Kumar Rao, Arun Kumar Arya, Krishna Kumar Dokania, Ved Prakash, Shakal Narayan Singh, Neeraj Kumar, Shiv Kumar, Vinay Pratap Singh, Pramod Kumar Singh, Vivek Kumar Singh, Rashmi Kumar, Aarti Kumar, Vishwajeet Kumar, Robinson Daniel Wammanda, Laila Hassan, Saraja Ahmodu Opaluwa, Ishaku Hassan, Aminu Shadrach Adamu, Bawa Ega, Daniel Efemena Atinaya, Fyezah Jehan, Imran Nisar, Benazir Baloch, Dania Omer Ansari, Kiran Lalani, Najeeb Rehman, Azhar Raza, Tooba Ahmed Alvi, Salman Osmani, Aneeta Hotwani, Erum Salman, Suneeta Namdave, Muhammad Hanif, Nasir Saleem Saddal, Jamal Raza, Syed Rehan Ali, Shahid Raza, Muhammad Tofique, Mashal Khan, Wajid Hussain, Muhammad Hayat Bozdar, Mahmood Shaikh, Musarat Ayaz, Sumaira Wajid, Serajunissa Syed, Muhammad Naveed, Hassan Abdul Jabbar, Azeem Khan, Karim Manji, Christopher R Sudfeld, Rodrick Kisenge, Nahya Salim, Sarah Somji, Mohamed Kheri Bakari, Fatimah Dhallah, Fred Maleko, Kristina Lugangira, Veneranda M Ndensangia, Christopher P Duggan, Vanessa Thorsten, Elizabeth McClure, Norman Goco, David Plotner, Barbara Do, Norma Pugh, Melissa Page, Sachiyo Yoshida, Shamim A Qazi, Rajiv Bahl, Yasir Bin Nisar

## Abstract

**Background:**

Research has shown low mortality in young infants with a single low-mortality-risk possible serious bacterial infection (PSBI) sign. Outpatient treatment of young infants (age <2 months) with a single low-mortality-risk PSBI sign might be as effective and safe as hospitalisation. Outpatient treatment overcomes the challenges of hospitalisation and improves access in low-resource settings. Our aim was to assess clinical outcomes in patients with one low-mortality-risk PSBI sign treated as outpatients compared with inpatient treatment.

**Methods:**

We did an open-label, multicentre, two-arm, randomised controlled trial at seven sites across Bangladesh, Ethiopia, India, Nigeria, Pakistan, and Tanzania. Young infants presenting to study hospitals with one of three low-mortality-risk PSBI signs (ie, fast breathing if age <7 days, body temperature ≥38°C, or severe chest indrawing) were randomly assigned (1:1) to the outpatient treatment group (2-day injectable gentamicin plus 7-day oral amoxicillin) or the inpatient treatment group (7-day injectable ampicillin plus gentamicin, with supportive care). The primary outcome was poor clinical outcome, which was a composite of any one of the following: death, critical illness, signs of other serious infections, new PSBI signs on days 2, 4, 8, and 15 or persistence of the presenting sign on day 8 after randomisation. We evaluated superiority and non-inferiority using the Farrington–Manning score test. The trial is registered with the ISRCTN Registry (ISRCTN44033252).

**Findings:**

Between June 24, 2021, and April 26, 2024, 7001 young infants were enrolled and randomly assigned to the outpatient treatment group (n=3501) or the inpatient treatment group (n=3500), and were part of the intention-to-treat (ITT) population. Poor clinical outcomes occurred in 269 (7·7%) of 3501 outpatients and 272 (7·8%) of 3500 inpatients in the ITT analysis (risk difference –0·0009 [95% CI –0·0134 to 0·0116]; p=1·0000 for superiority analysis). Deaths were significantly lower in the outpatient group (nine [0·3%] of 3501) than in the inpatient group (23 [0·7%] of 3500; risk difference –0·0040 [–0·0072 to –0·0008]). In the per-protocol analysis, outpatient treatment (266 [7·7%] of 3455) was non-inferior to inpatient treatment (269 [7·9%] of 3416) for poor clinical outcomes (risk difference –0·0018 [–0·0144 to 0·0109]; p=0·0012 for non-inferiority). Apart from deaths, there were no treatment-related serious adverse events.

**Interpretation:**

Outpatient treatment (gentamicin injection and oral amoxicillin) for infants with a single low-mortality-risk PSBI sign was non-inferior to standard inpatient treatment, with significantly lower mortality in the outpatient treatment group.

**Funding:**

Bill and Melinda Gates Foundation.

## Introduction

Countries in south Asia and sub-Saharan Africa contribute substantially to the global burden of newborn deaths, accounting for 1·9 million (83%) of the 2·3 million neonatal deaths worldwide in 2022.^1^ Neonatal infections remain a crucial challenge in these regions, contributing to up to 37% of neonatal deaths.^2^ WHO has developed recommendations for the management of suspected sepsis in young infants up to age 2 months, characterised as a possible serious bacterial infection (PSBI).^3^ WHO's existing protocol for Integrated Management of Childhood Illness (IMCI) recommends hospitalisation with injectable antibiotics for at least 7 days for young infants (age <2 months) with PSBI (appendix 1 p 1).^3^, ^4^ However, when a referral is not feasible, IMCI alternatively recommends outpatient care with a simplified antibiotic regimen (appendix 1 p 1) for those subclassified as having a clinical severe infection.^3^, ^5^ The feasibility of this outpatient therapy has been shown in multiple African and Asian countries.^6^

**Research in context**

**Evidence before this study**

We systematically searched PubMed, Embase, and Cochrane Library databases to identify studies in any language on outpatient versus inpatient management of possible serious bacterial infection (PSBI) in young infants, and any differences in treatment strategies based on mortality risk. Our search terms included “possible serious bacterial infection”, “PSBI”, “young infants”, “outpatient treatment”, “inpatient treatment”, and “mortality risk”, covering studies published from Jan 1, 2010, to Nov 30, 2024. We identified several trials, notably the AFRINEST and SATT trials, which suggested that some young infants can be safely managed as outpatients. However, these studies were limited to infants with various clinical severe infection signs, without stratifying by low-mortality-risk PSBI signs alone. Observational studies indicated a three-fold higher mortality rate for infants with clinical severe infection signs in the inpatient treatment group than in the outpatient treatment group. However, these studies did not have randomised comparisons and were prone to selection bias. Based on this background, we identified the need for a randomised trial to compare clinical outcomes of outpatient versus inpatient care in young infants with single low-mortality-risk PSBI signs.

**Added value of this study**

Our study is the first randomised controlled trial comparing outpatient and inpatient care in young infants with a single low-mortality-risk PSBI sign, done across multiple low-income and middle-income countries in south Asia and sub-Saharan Africa. Our findings show that outpatient treatment using gentamicin and oral amoxicillin is non-inferior to inpatient care in preventing poor clinical outcomes. Additionally, the outpatient group had a significantly lower mortality rate, suggesting potential reduced exposure to health-care-acquired infections. This study thus offers strong evidence supporting outpatient management as a viable and potentially safer option for specific low-mortality-risk PSBI cases, which could transform current treatment approaches.

**Implications of all the available evidence**

Together with previous studies, our findings support updating WHO PSBI management guidelines to recommend outpatient treatment as a primary option for infants with a single low-mortality-risk PSBI sign, even where hospital referral is feasible. This approach could optimise resource allocation, improve caregiver acceptance, and reduce health-care costs. The lower mortality observed with outpatient care also suggests potential benefits in reducing hospital-acquired infections and antimicrobial resistance in low-resource settings. Future research could further explore optimising the treatment location for infants with higher-risk PSBI signs and investigate point-of-care diagnostics to aid early differentiation of bacterial versus viral infections.

In low-income and middle-income countries (LMICs), where an estimated 11% of all young infants develop PSBI,^7^ only about 19% are referred to a hospital for treatment.^6^ Hospitalisation poses multiple challenges for families, health systems, and infants. Families of sick young infants encounter barriers related to availability, cost, access, and quality of services.^8^, ^9^, ^10^ From a health system perspective, hospitalising all young infants with PSBI would overburden these resource-constrained systems and expose infants to a risk of health-care-acquired infections (HAIs).^11^ Moreover, infants might leave or be discharged from the hospital before completing the treatment due to financial constraints, further laboratory tests and procedures, or quality of care issues without adequate follow-up care.^8^, ^12^, ^13^, ^14^ Data from several LMICs show that coverage for PSBI management on an outpatient basis was much higher (31–94%) than for inpatient management (2–34%).^6^ These factors underscore the need to evaluate safe outpatient options that align with family and health system constraints.

Recent findings suggest that outpatient treatment might be safer or at least equivalent to hospital care in a subgroup of infants with PSBI. Secondary analysis of the AFRINEST study^15^, ^16^ showed that the mortality rate for young infants categorised as having clinical severe infection and treated as inpatients was three times higher (6·5%) than those receiving outpatient treatment (1·9%; appendix 1 p 12).^17^ The secondary analysis further identified three clinical severe infection signs with a low mortality risk when presenting alone—fast breathing (≥60 breaths per min in infants aged 0–6 days) with a mortality rate of 2%, body (axillary) temperature ≥38°C with a mortality rate of 0·8%, or severe chest indrawing with a mortality rate of 0·9%. Although the population seeking hospital care might differ from that receiving outpatient care and, therefore, is not directly similar, these findings show the potential of treating sick young infants presenting with any of these three low-mortality-risk signs at the outpatient level without referral to a hospital.

We aimed to optimise the place of treatment for sick young infants with a single low-mortality-risk sign of PSBI^17^ by experimentally comparing the clinical outcomes in outpatient versus inpatient treatment. We hypothesised that these sick young infants managed as outpatients would have superior or non-inferior outcomes to those receiving inpatient care.^18^

## Methods

### Study design

We did an open-label, multicentre, two-arm, individual-level, randomised controlled trial in seven sites in six LMICs (ie, Bangladesh, Ethiopia, India [two sites], Nigeria, Pakistan, and Tanzania). We enrolled participants at multiple hospitals at each site. The study received ethical approval from the WHO Ethics Review Committee and relevant local ethics committees at each site (appendix 1 pp 2–4). We published the trial methodology details previously.^18^ The study is registered with the ISRCTN Registry (ISRCTN44033252).

### Participants

Young infants were considered eligible for screening if they were younger than 2 months, lived in an area where they could be followed up for at least 15 days, and presented at study hospitals with any one of the following single low-mortality-risk signs of PSBI (body axillary temperature ≥38°C, severe chest indrawing, or fast breathing of ≥60 breaths per min in infants aged 0–6 days).^17^ Young infants were considered eligible for enrolment only after agreement on the assessment of the eligibility criteria between the screening and enrolment team and the hospital physician. We excluded young infants if they presented with other signs of PSBI, low birthweight (<2 kg if younger than 10 days), severe malnutrition, other serious illnesses, appearance of low-risk signs within the first 24 h of life, or current or previous participation in any study (appendix 1 p 5). Written informed consent was obtained in the local language from caregivers before screening and subsequently for enrolment of eligible infants.

### Randomisation and masking

Participants were randomly assigned (1:1) to two groups: the outpatient treatment group (intervention) or the inpatient treatment group (control) using a block randomisation scheme stratified by study site and hospital. A non-study staff member at WHO generated computerised randomisation lists with unique participant identification numbers for each study hospital using random block sizes of 2, 4, 6, and 8. The data coordination centre converted participants’ unique identification numbers from the randomisation list of each hospital into encrypted QR codes, which were printed sequentially on labels and shipped to each site. After obtaining consent for enrolment, the screening and enrolment team obtained the treatment allocation by scanning the next available encrypted QR code label into an electronic data capture application. Upon scanning the QR code, the system validated that the QR code had not been scanned or used previously and no longer allowed the user to back up or scan another code unless the one scanned was not accepted as valid. This prevented altering the treatment group after scanning the QR code. Additionally, the treatment group's assignment information in the QR code was encoded, and no humans would have been able to interpret the randomisation treatment that it contained. Masking of participants, hospital clinical teams, the screening and enrolment team, and the study treatment documentation team was not possible because the location of treatment for the intervention and control groups was different. However, the risk of bias was mitigated by having an independent outcome assessment team that was not present during screening, enrolment, or treatment documentation.

### Procedures

Young infants in the intervention group received outpatient treatment with gentamicin (20 mg/mL or 40 mg/mL), injected intramuscularly once daily for 2 days, and amoxicillin (250 mg dispersible tablet, 125 mg dispersible tablet, or 125 mg in 5 mL suspension), taken orally twice daily for 7 days (table 1).^3^ The first dose of injectable gentamicin was administered in the hospital at the time of enrolment (day 1), along with the first dose of oral amoxicillin, which was provided by caregivers under the supervision of the screening and enrolment team. Caregivers were provided the full course of oral amoxicillin for the 7 days of treatment with instructions to administer it, including repeating the dose if the infant vomited part or all of the antibiotic within 20 min of administration. They were asked to return to the hospital's outpatient department on the following day for the second dose of gentamicin. They were counselled about the timing of follow-up visits, danger signs in young infants, and when to seek care. During the follow-up visit at the outpatient clinic on day 2, the hospital physician assessed infants for the presence of any adverse event or worsening of illness and, if found, managed them as per routine practice.

**Table 1:** Treatment medications, route, and dosage for the outpatient treatment group (intervention)^3^ by weight of infant

	**Gentamicin (intramuscular, 20 mg/mL)**	**Gentamicin (intramuscular, 40 mg/mL)**	**Amoxicillin (oral dispersible tablet, 250 mg per dose)**	**Amoxicillin (oral dispersible tablet, 125 mg per dose)**	**Amoxicillin (oral suspension, 125 mg/5 mL per dose)**
1·5–2·4 kg	0·4 mL per dose	0·2 mL per dose	Half a tablet	One tablet	5 mL
2·5–3·9 kg	0·8 mL per dose	0·4 mL per dose	Half a tablet	One tablet	5 mL
4·0–5·9 kg	1·2 mL per dose	0·6 mL per dose	One tablet	Two tablets	10 mL

Infants in the control group received the WHO-recommended inpatient antibiotic treatment consisting of ampicillin (250 mg/1·5 mL), injected intramuscularly two to four times daily (depending on age), and gentamicin (20 mg/mL or 40 mg/mL), injected intramuscularly once daily, along with supportive care for at least 7 days (table 2).^4^ Infant care services in participating hospitals were strengthened through training and operationalising standard operating procedures in compliance with WHO's quality of care.^4^ Hospital clinical teams were oriented on the recommended study treatment protocols. All infants enrolled in the study received antibiotics using weight band dosing (table 2). WHO procured study drugs for both treatment groups centrally.

**Table 2:** Treatment medications, route, and dosage for the inpatient treatment group (control)^4^ by weight of infant

	**Gentamicin (intramuscular, 20 mg/mL)**	**Gentamicin (intramuscular, 40 mg/mL)**	Ampicillin (intramuscular, 250 mg/1·5 mL)*
1·5–2·4 kg	0·4 mL per dose	0·2 mL per dose	0·8 mL per dose
2·5–3·9 kg	0·8 mL per dose	0·4 mL per dose	1·2 mL per dose
4·0–5·9 kg	1·2 mL per dose	0·6 mL per dose	1·5 mL per dose

* Depending on the age of the child, for the hospitalised child, twice a day for the first week of life, three times a day for ages 2–4 weeks, and then four times a day for ages 4 weeks and older.

The study treatment documentation team visited young infants in both study groups at 2, 4, and 8 days after randomisation to document the treatment received throughout the 7 days*.* Treatment received in hospital wards or outpatient facilities was recorded from the hospital records for both groups. Home treatment was documented using caregiver recall, counting the remaining amoxicillin dispersible tablets or measuring the amoxicillin suspension.

The independent outcome assessments assessed study outcomes through visits either at the hospital or home, depending on the location of the infant, on days 2, 4, 8, and 15 after randomisation. During days 2, 4, and 8, the independent outcome assessment did a complete assessment of PSBI and other signs of infection and recorded other outcome-related information, and on day 15, only the vital status was recorded.

Training was harmonised across sites through a centrally coordinated process led by WHO. Study teams received in-person training on PSBI assessment based on the IMCI protocol,^3^ using standardised standard operating procedures for clinical and study procedures. Quarterly standardisation exercises with video-based assessments and central feedback ensured consistency across sites. We repeated the training as needed until WHO monitored and the coordinating team confirmed standardisation. Sites had regular refresher training for the screening and enrolment team and the independent outcome assessment team. WHO provided ongoing oversight through monthly reviews and annual site visits.

All sites used an electronic data capture application to standardise data collection and synchronise encrypted data with site-specific cloud servers in real time. Sites stored original data on password-protected servers, which were anonymised and synced monthly with an independent data coordination centre that reported directly to WHO, ensured data integrity, and sent monthly data quality reports to sites for issue resolution. Sites securely stored and limited access to consent forms and physical documents linking personal data to participant identifiers to maintain confidentiality. Data were centrally analysed.

An independent data and safety monitoring board was established to monitor the trial and review safety outcomes. The data and safety monitoring board did quarterly reviews of safety outcomes and prespecified interim analyses for efficacy at 25%, 50%, and 75% of total enrolment, with treatment allocation masked. For safety monitoring, stopping decisions were based on a significance level of α=0·05, with death as the primary safety outcome and serious adverse events and critical illness as secondary safety endpoints. For efficacy and futility, formal stopping boundaries were applied using the O’Brien–Fleming approach. The corresponding nominal significance thresholds were p=0·00005 (first interim), p=0·0039 (second interim), and p=0·0184 (third interim), with a final analysis threshold of p=0·0412. The primary efficacy outcome was a composite of poor clinical outcomes. However, the final critical threshold was not applied, and the sample size was not adjusted to account for the interim analyses. Additionally, a technical advisory group was established, which met once before the study started and thereafter yearly to review the study protocol, procedures, and tools, and provide technical guidance on the study implementation.

### Outcomes

The primary outcome of the study was a hierarchical composite indicator called poor clinical outcome defined as: (1) death between randomisation and day 15 from randomisation; (2) the presence of any sign of critical illness (no movement at all, unable to feed at all, or convulsions) on day 2, 4, or 8 from randomisation; (3) any sign suggestive of another serious infection (eg, meningitis, or bone or joint infection on day 2, 4, or 8 from randomisation; (4) presence of any new sign (same sign as screening at day 8 after disappearing on day 4, or a different sign from screening on day 4 or 8) of clinical severe infection on day 4 or 8 from randomisation; or (5) persistence of the presenting sign on day 8 (same sign at screening, day 4, and day 8) from randomisation. Poor clinical outcome was coded as yes, no, or lost to follow-up or withdrew consent. In infants lost to follow-up or with withdrawn consent, no information was gathered about the infant's outcome after they were lost to follow-up or withdrew from the study. Secondary outcomes include the individual components of poor clinical outcome, specifically death, critical illness, another serious infection, any new clinical severe infection, and persistence of the presenting clinical severe infection sign.

Serious adverse events, including anaphylactic reactions, other allergic reactions, injection site infection, and diarrhoea with severe dehydration, were identified during follow-up visits at the hospital or home, or when families visited the hospital for care-seeking, or when families reported such events at any time to any study staff, which were then sent to the study physician for verification and confirmation. Hospital physicians managed adverse events per the local standard of care until resolution.

### Statistical analysis

The sample size assumed that 6% of young infants with a single low-mortality-risk sign of PSBI would have poor clinical outcomes after treatment in the control group.^15^, ^16^ For the superiority analysis, we required a sample size of 7000 infants to detect a 30% relative reduction in poor clinical outcome with an alpha error of 0·05 and 80% power. This sample size was sufficient to detect a non-inferiority margin of 1·8% at 90% power.^18^

Frequencies (with percentage), means (SD), and medians (IQR) of baseline characteristics, as applicable, were compared between outpatient and inpatient groups to verify that the randomisation was balanced. We did a superiority analysis using an intention-to-treat (ITT) approach to determine whether outpatient treatment resulted in a significantly lower rate of poor clinical outcomes than inpatient care, with a 30% relative reduction indicating that outpatient care is superior to inpatient care. We also did a non-inferiority analysis using a per-protocol approach to assess whether outpatient treatment was not worse than inpatient care, within a non-inferiority margin of 1·8%. We used the Farrington–Manning (1990) score test for superiority and non-inferiority analysis. Participants who were withdrawn or were lost to follow-up and for whom poor clinical outcome data were available before censoring were included in the ITT population. The per-protocol population excluded participants who were lost to follow-up, withdrew consent, did not initiate study medication, or were ineligible for enrolment and accidentally randomised.

Poor clinical outcome rates were compared between treatment groups for all sites combined for both the superiority and non-inferiority analyses. We calculated the risk differences with 95% CIs and relative risk with 95% CI between the treatment groups. Unadjusted and adjusted risk difference with 95% CI for the poor clinical outcome were estimated with the generalised linear models for binary data. The models were specified with a binomial distribution and an identity link function, which allows direct estimation of risk differences. The unadjusted model included only the treatment group as a fixed effect. The adjusted model included both treatment group and site as fixed effects. We did the prespecified secondary analysis similarly for each component of the hierarchical composite indicator. Infants exhibiting multiple components of the composite indicator were categorised under the most severe component.

For treatment compliance measurements, in the outpatient group, we categorised infants who received two doses of injection gentamicin and 14 doses of oral amoxicillin with no other antibiotics during the 7 days of treatment as received all medication, and those who received two doses of injection gentamicin along with at least ten doses of oral amoxicillin and no other antibiotics during the 7 days of treatment were categorised as received at least 80% of the medication. In the inpatient group, we categorised infants who received all daily doses of injection ampicillin and injection gentamicin with no other antibiotics during the first 7 days of treatment as received all medication, and those who received the complete daily dose of injection ampicillin and injection gentamicin for at least 5 days without any other antibiotics were categorised as received at least 80% of the medication. We used the χ^2^ test to compare the adherence, loss to follow-up or withdrawal from the study, and serious adverse events, apart from death, between the two study groups. We used Stata version 18.0 and SAS version 9.4 accompanied by statistical analysis plan version 1.0 (appendix 1 pp 6–45).

### Role of the funding source

The funder had no role in study design, data collection, data analysis, data interpretation, or writing of the report.

## Results

**Figure 1:**
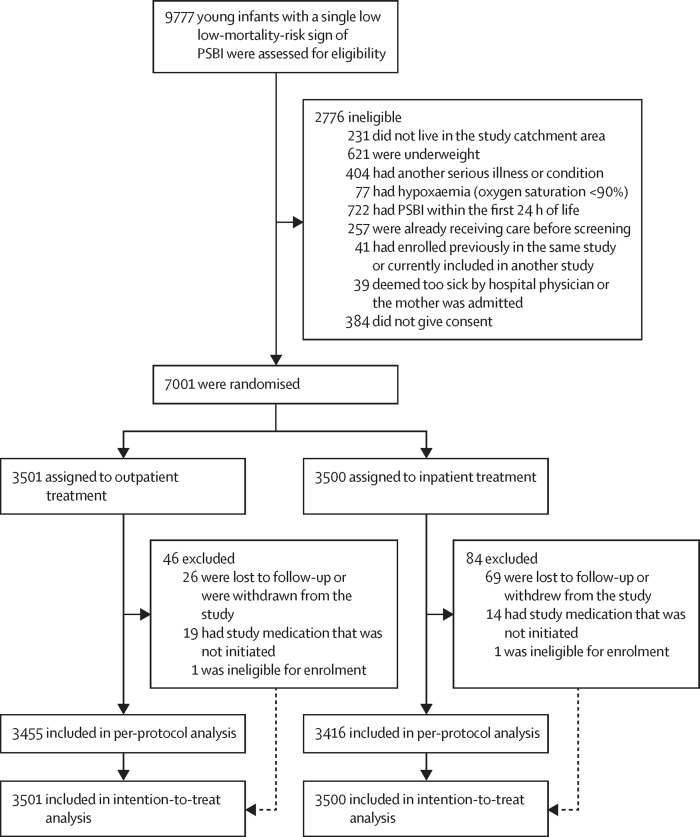
Trial profile PSBI=possible serious bacterial infection.

Between June 24, 2021, and April 26, 2024, 9777 young infants with a single low-mortality-risk PSBI sign were screened (figure 1). Of these, 7385 (75·5%) were eligible. Among the eligible infants, 7001 were enrolled after parental or caregivers consent and randomly assigned to the outpatient treatment group (n=3501) or the inpatient treatment group (n=3500), contributing to the ITT population for a superiority analysis. After randomisation, 130 (1·9%) of 7001 infants were excluded: 95 (73·1%) who were lost to follow-up or whose caregivers withdrew consent; 33 (25·4%) who did not initiate study medication; and two (1·5%) who did not meet enrolment criteria but were randomly assigned (figure 1). Thus, the per-protocol population included 6871 (98·1%) of 7001 infants: 3455 in the outpatient treatment group and 3416 in the inpatient treatment group. Baseline characteristics, including age, weight, and single low-mortality-risk PSBI signs, were well balanced across treatment groups, indicating effective randomisation (table 3).

**Table 3:** Baseline characteristics of the intention-to-treat population and per-protocol population

	**Intention-to-treat population**		**Per-protocol population**	
	Outpatient treatment group (n=3501)	Inpatient treatment group (n=3500)	Outpatient treatment group (n=3455)	Inpatient treatment group (n=3416)
**Region**				
Africa	1568 (44·8%)	1556 (44·5%)	1547 (44·8%)	1519 (44·5%)
Asia	1933 (55·2%)	1944 (55·5%)	1908 (55·2%)	1897 (55·5%)
**Study site**				
Bangladesh	604 (17·3%)	601 (17·2%)	604 (17·5%)	597 (17·5%)
Ethiopia	268 (7·7%)	256 (7·3%)	259 (7·5%)	255 (7·5%)
India site 1	242 (6·9%)	246 (7·0%)	239 (6·9%)	241 (7·1%)
India site 2	526 (15·0%)	533 (15·2%)	513 (14·8%)	513 (15·0%)
Nigeria	600 (17·1%)	600 (17·1%)	589 (17·0%)	565 (16·5%)
Pakistan	561 (16·0%)	564 (16·1%)	552 (16·0%)	546 (16·0%)
Tanzania	700 (20·0%)	700 (20·0%)	699 (20·2%)	699 (20·5%)
**Sex**				
Male	2062 (58·9%)	2184 (62·4%)	2031 (58·8%)	2131 (62·4%)
Female	1439 (41·1%)	1316 (37·6%)	1424 (41·2%)	1285 (37·6%)
**Age, days**				
1–6	969 (27·7%)	1032 (29·5%)	956 (27·7%)	1017 (29·8%)
7–28	1140 (32·6%)	1145 (32·7%)	1125 (32·6%)	1112 (32·6%)
29–59	1392 (39·8%)	1323 (37·8%)	1374 (39·8%)	1287 (37·7%)
**Weight-for-age Z score**				
Less than −2	637 (18·2%)	674 (19·3%)	622 (18·0%)	659 (19·3%)
−2 to less than −1	1123 (32·1%)	1121 (32·0%)	1110 (32·1%)	1093 (32·0%)
−1 to 0	1199 (34·2%)	1114 (31·8%)	1183 (34·2%)	1081 (31·6%)
More than 0	542 (15·5%)	591 (16·9%)	540 (15·6%)	583 (17·1%)
**Single low-mortality-risk possible serious bacterial infection sign**				
Severe chest indrawing	1369 (39·1%)	1436 (41·0%)	1349 (39·0%)	1387 (41·0%)
High body temperature of ≥38°C	1819 (52·0%)	1725 (49·3%)	1799 (52·1%)	1696 (49·6%)
Fast breathing in infants aged 1–6 days	313 (8·9%)	339 (9·7%)	307 (8·9%)	333 (9·7%)

Data are n (%).

**Figure 2:**
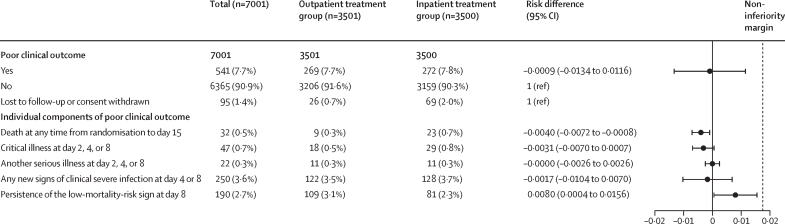
Poor clinical outcome of the intention-to-treat population Data are n (%), unless stated otherwise. The black dotted line represents the non-inferiority margin.

**Figure 3:**
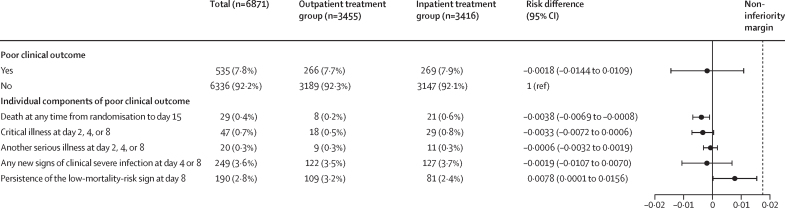
Poor clinical outcome of the per-protocol population Data are n (%), unless stated otherwise. The black dotted line represents the non-inferiority margin.

In the ITT population, by day 15, poor clinical outcomes occurred in 269 (7·7%) of 3501 infants in the outpatient treatment group and 272 (7·8%) of 3500 infants in the inpatient treatment group, with no significant difference between groups (risk difference –0·0009 [95% CI –0·0134 to 0·0116]; p=1·0000 for superiority; figure 2; appendix 1 p 46). In the per-protocol population, outpatient treatment was determined to be non-inferior to inpatient care in terms of poor clinical outcomes: 266 (7·7%) of 3455 infants in the outpatient group versus 269 (7·9%) of 3416 in the inpatient group (risk difference –0·0018 [95% CI –0·0144 to 0·0109]; p=0·0012 for non-inferiority; figure 3; appendix 1 p 47). The relative risk for ITT was 0·99 (95% CI 0·84 to 1·16) and for the per-protocol population was 0·98 (95% CI 0·83 to 1·15).

The components of the composite outcome are reported in hierarchical order. In the ITT population, mortality by day 15 in the outpatient treatment group (nine [0·3%] of 3501) was significantly lower than the inpatient treatment group (23 [0·7%] of 3500; risk difference –0·0040 [95% CI –0·0072 to –0·0008]; figure 2). Similarly, in the per-protocol population, mortality by day 15 in the outpatient treatment group (eight [0·2%] of 3455) was significantly lower than in the inpatient treatment group (21 [0·6%] of 3416; risk difference –0·0038 [95% CI –0·0069 to –0·0008]; figure 3). However, a significantly lower proportion of young infants in the inpatient group showed persistence of the original low-mortality-risk sign on day 8 compared with those in the outpatient group in both the ITT group (risk difference 0·0080 [95% CI 0·0004 to 0·0156]; figure 1) and the per-protocol group (risk difference 0·0078 [95% CI 0·0001 to 0·0156]; figure 2). The occurrence of component of poor clinical outcome was similar in both groups.

Adherence to the prescribed treatment was high, with 3178 (90·8%) of 3501 outpatients and 3070 (87·7%) of 3500 inpatients receiving all doses, and 3240 (92·5%) outpatients and 3172 (90·6%) inpatients receiving at least 80% of study antibiotics (table 4). The occurrence of serious adverse events, apart from deaths, was similar in the outpatient (five [0·1%]) and inpatient (one [<0·1%]) groups, supporting the safety of the outpatient protocol. A significantly lower number of infants were lost to follow-up or withdrew from the study in the outpatient treatment group (26 [0·7%]) than those in the inpatient treatment group (69 [2·0%]; table 4; appendix 1 p 48). For comparison of baseline characteristics between those who were lost to follow-up or withdrew and those who were not see appendix 1 (p 49).

**Table 4:** Adherence with the study medication, serious adverse events (excluding deaths), and loss to follow-up or withdrawal from the study of the intention-to-treat population

		**Outpatient treatment group (n=3501)**	**Inpatient treatment group (n=3500)**
Adherence with study medication		..	..
	Study medication initiated	3469 (99·1%)*	3425 (97·9%)†
	Received all the study medication	3178 (90·8%)	3070 (87·7%)
	Received at least 80% of the study medication	3240 (92·5%)	3172 (90·6%)
Serious adverse events, excluding deaths		5 (0·1%)‡	1 (<0·1%)§
Lost to follow-up or withdrawal from the study		26 (0·7%)	69 (2·0%)

* In 18 infants, the hospital physician initiated other antibiotics after randomisation, whereas this information was missing in 14 infants.

† In ten infants, the hospital physician initiated other antibiotics after randomisation, whereas this information was missing in 65 infants.

‡ Four infants had diarrhoea, and one developed a rash; none of these were considered treatment-related adverse events.

§ Infant had diarrhoea; this was not considered a treatment-related adverse event.

## Discussion

In this large, multicentre, randomised controlled trial, we showed that the outpatient management of young infants with a single low-mortality-risk PSBI sign, although not superior, is non-inferior to inpatient care in averting poor clinical outcomes. Notably, the risk of mortality, the worst outcome in the hierarchical composite poor clinical outcome indicator, was significantly lower in the outpatient treatment group than in the inpatient treatment group. Although less critical, a significantly lower proportion of young infants in the inpatient treatment group showed persistence of the original low-mortality-risk sign on day 8 compared with those in the outpatient treatment group. The occurrence of other severe adverse events, apart from deaths, was similar. Adherence to antibiotic treatment regimens was high in both treatment groups, and notably, there was significantly lower loss to follow-up or withdrawal in the outpatient group.

Our findings support the use of simplified antibiotic regimens in the outpatient management of young infants with a single low-mortality-risk PSBI sign in resource-limited settings, in concordance with previous studies, such as AFRINEST^15^ and SATT,^19^, ^20^ which found that treatment with intramuscular gentamicin for 2 days and oral amoxicillin for 7 days was as effective as a 7-day regimen of parenteral antibiotics. Similar to AFRINEST and SATT, antibiotic adherence in our study was high in the outpatient setting.^15^, ^19^, ^20^ The observational finding of AFRINEST data of higher mortality risk in infants with clinical severe infection treated as inpatients versus outpatients formed the basis for the hypothesis in this experimental study.^17^ Our findings of non-inferiority of outpatient treatment compared with inpatient care regarding poor clinical outcomes and lower risk of mortality in the outpatient group are consistent with the AFRINEST findings.^15^ It is important to consider that our trial only enrolled infants with single low-mortality-risk signs of PSBI. In contrast, AFRINEST and SATT enrolled infants with single and multiple PSBI signs and signs associated with greater mortality risk (ie, stopped feeding well, low body temperature, and movement only when stimulated).^15^, ^19^, ^20^

Our results suggest that outpatient treatment is a safe and feasible alternative for infants with a single low-mortality-risk PSBI sign, helping alleviate the demand for hospital resources without compromising care quality. Several factors might explain these findings. First, inpatient treatment carries an inherent risk of HAIs. Although all sites adopted measures to strengthen inpatient care quality, maintaining consistently high standards in resource-constrained settings with high patient loads and intermittent staff shortages, equipment, diagnostics, and supplies remains challenging. PSBI is a syndromic diagnosis based on various clinical signs, but since our trial population consisted of infants with a single low-mortality-risk sign, the relative benefit of inpatient care might be inadequate compared with its risks. We observed lower mortality in the outpatient group, possibly due to less invasive treatment and reduced exposure to hospital-based pathogens, reducing the risk of HAIs.^21^, ^22^ Adherence to the prescribed antibiotic treatment was high in the outpatient and inpatient groups. The simplified antibiotic regimen used for outpatient management will likely have had a facilitatory role in high adherence. Finally, the oral amoxicillin dose used in our study results in adequate serum concentrations with a 12-h dosing interval,^23^ making it a safe replacement for parenteral penicillin or ampicillin.

These findings have several implications for national policy makers, newborn and child health programmes, and families. First, they can inform the refinement of the current WHO guidelines for managing PSBI in LMIC settings. The 2015 WHO guidelines for the management of PSBI in young infants offered outpatient management with simplified antibiotic regimens as a secondary alternative to hospital-based care in cases where referral was not feasible.^5^ Although referral was feasible in our study, we found that outpatient management was non-inferior to inpatient care in infants with a single low-mortality-risk PSBI sign, suggesting that outpatient care can be recommended as the primary strategy for the management of these infants rather than as a secondary alternative to inpatient care. This approach would reduce the burden on hospitals while improving caregiver acceptance of treatment by minimising disruption to their lives and affording greater flexibility to attend to their household, work, or other responsibilities.^10^, ^24^, ^25^ Moreover, outpatient management of these infants might provide more equitable access to treatment, as we found in our trial (90·8% in the outpatient group and 87·7% in the inpatient group received all the study medication).

The second key implication is that outpatient treatment can reduce costs for health systems and families, potentially improving treatment availability and adherence in LMICs. In sub-Saharan Africa, the cost estimate for managing neonatal sepsis is between US$10 billion and $469 billion annually.^26^ Outpatient treatment is likely to substantially reduce the financial burden on families by eliminating costs associated with hospital registration, admission and bed fees, medicine costs, food, transport, and loss of wages due to stay in the hospital.^12^, ^13^, ^27^ Mean costs of hospitalisation and costs to households per episode of neonatal sepsis were estimated at $308 and $50, respectively, in Mozambique and $684 and $52 in South Africa.^27^ Outpatient management of PSBI, when referral is not feasible, compared with inpatient treatment, has been reported to be cost-effective in several countries.^28^, ^29^ Shifting care of young infants with a single low-mortality-risk PSBI sign to outpatient settings allows for a more efficient allocation of health-care resources,^30^ with hospitalisation focused on care of more critically ill infants at a higher risk of mortality.^31^ We will present the specific cost-related data from our study separately.

Other essential implications of the outpatient-based treatment of PSBI are the potential for a lower risk of HAIs and reduced pressure on antimicrobial resistance (AMR). HAIs are a major global problem that disproportionately affect LMICs, with frequencies of HAIs reported to be two to three times higher than in high-income countries.^21^ Estimated pooled prevalence of mortality resulting from HAIs in sub-Saharan Africa was 22%.^22^ HAIs, often caused by antibiotic-resistant bacteria, are associated with the length of inpatient stay and invasive interventions in hospitalised newborns and can result in severe morbidities, high case-fatality rates, and costs.^32^, ^33^, ^34^, ^35^ In 2019, nearly 5 million deaths globally were estimated to be associated with AMR, with sub-Saharan Africa and south Asia having the highest rates of AMR-associated deaths.^36^ With the only invasive intervention in the outpatient group of this study being intramuscular gentamicin on the first 2 days of treatment at a facility, the risk of HAIs in these infants would be very low. A simplified outpatient antibiotics regime with high compliance and completion rates and lower risks of AMR and HAIs would likely remain effective longer than hospital-based treatments.

Although the findings of this study are encouraging, successful implementation and scale-up will require careful consideration of potential system-level challenges. These might include variations in health system capacity, workforce availability, and the functionality of referral systems across different settings. Implementation research is needed to identify context-specific barriers and facilitators. Moreover, uptake will likely depend on changing the perceptions and practices of health-care providers and securing the support of policy makers and other stakeholders. Tailored strategies and policy engagement at the national level will be crucial for sustainable integration into routine care.

Our study had several strengths. It was a prospective, multicentre, multicountry, randomised trial in sub-Saharan African and south Asian populations with a large sample size representing diverse populations and health systems, enhancing the generalisability of our findings. Baseline characteristics were balanced across outpatient and inpatient treatment groups, signifying effective randomisation. We implemented the study to a high standard, with central and site-specific coordination and oversight. Assessment of PSBI signs was standardised across sites, enhancing the reliability of the findings.^18^ Additionally, we ensured that all study hospitals followed standards for neonatal care in line with the WHO guidance.^4^

Our study also had limitations. First, disruption due to the COVID-19 pandemic caused delays and interruptions at the start of data collection, resulting in adjusted timelines across different locations. Second, the trial was not entirely masked. The treatment allocation was apparent to care providers and parents in outpatient and inpatient groups. However, the independent outcome assessment remained masked to the presenting clinical signs at enrolment and other patient support activities. Third, being a multicountry study, there were differences in the organisation and functionality of the health system structures and facilities. Country-specific issues, such as security concerns and natural disasters (eg, floods, which might have affected the consistency of service provision), compounded the issue. Additionally, we used clinical features alone to diagnose PSBI, without any laboratory data to assess the presence and aetiology of infection. However, most hospitals with laboratory services treat neonates with suspected sepsis empirically based on clinical signs.^37^ Despite sophisticated laboratory services, a large, multicentre study of aetiology in PSBI from south Asia showed that identifying bacterial infection among young infants is challenging.^38^

Empirical data does not guide the appropriate duration of treatment for PSBI; therefore, it is crucial to evaluate the optimal duration of hospitalisation and whether sick young infants with higher mortality risk signs of PSBI can be discharged to complete antibiotic therapy at home. Our study team also did a trial among infants with moderate-mortality-risk signs of PSBI,^39^ and the results are forthcoming. It is also imperative to identify suitable point-of-care tests to differentiate between bacterial and viral causes of PSBI to rationalise antibiotic use further, lessen the pressure on antibiotics, and improve outcomes.^40^ Finally, research is needed to explore other simple measures beyond infection control that could make hospitalisation safer for young infants.

In conclusion, treatment of young infants with a single low-mortality-risk PSBI sign was equally effective when they were managed as outpatients with injectable gentamicin for 2 days and oral amoxicillin for 7 days compared with inpatient management with injectable ampicillin and gentamicin for 7 days. The risk of infant death was lower in the outpatient treatment group than in the inpatient treatment group. Adherence to antibiotic treatment regimens was high in both treatment groups, and notably, there was significantly lower loss of follow-up or withdrawal in the outpatient group. Our findings support a policy review of existing WHO guidelines for PSBI management, recommending outpatient care as the primary approach for low-mortality-risk PSBI cases. Such a shift towards outpatient care for low-mortality-risk PSBI cases is a primary recommendation that could enhance equitable access and coverage of PSBI treatment, decrease potential HAIs and AMR, reduce health-care costs for both the health system and families, and ultimately improve care outcomes in resource-limited settings.

### PSBI Study Group

### Contributors

### Equitable partnership declaration

### Data sharing

## Declaration of interests

YBN and SY are staff members of WHO. All other authors declare no competing interests. The authors alone are responsible for the views expressed in this Article, and they do not necessarily represent the views, decisions, or policies of the institutions with which they are affiliated.

## Supplementary Materials

Supplementary appendix 1

Equitable Partnership Declaration
