# Supplementary material for: Inpatient versus outpatient management of young infants with a single low-mortality-risk sign of possible serious bacterial infection in sub-Saharan Africa and south Asia: an open-label, multicentre, two-arm, randomised controlled trial
Source: Lancet Glob Health. 2025 Oct 15;13(11):e1892–902. doi: 10.1016/S2214-109X(25)00243-8 (PMC12535820; doi:10.1016/S2214-109X(25)00243-8)
Supplement: Supplementary appendix 1 [file mmc1.pdf]

THE LANCET
Global Health

Supplementary appendix 1

This appendix formed part of the original submission and has been peer reviewed. We post it as supplied by the authors.

Supplement to: PSBI Study Group. Inpatient versus outpatient management of young infants with a single low-mortality-risk sign of possible serious bacterial infection in sub-Saharan Africa and south Asia: an open-label, multicentre, two-arm, randomised controlled trial. *Lancet Glob Health* 2025; **13:** e1892–902.

Appendix 1

Supplementary material

Contents

Table SM1: Classification and management of young infants with any sign of possible serious bacterial infection (PSBI)........ 1

Table SM2: Ethics approvals, participating hospitals, dates for the initiation and end of enrolment by study site........ 2

STATISTICAL ANALYSIS PLAN FOR THE PRIMARY ANALYSIS........ 6

Table SM3: Poor clinical outcome using intent-to-treat population, adjusted by THE STUDY site (n=7001)........ 46

Table SM4: Poor clinical outcome using per-protocol population, adjusted by THE STUDY site (n=6871)........ 47

Table SM5: Reasons for loss to follow-up/withdrawal from the study by treatment arm (n=95)........ 48

Table SM6: Comparison of baseline characteristics of young infants who were lost to follow-up/withdrawal and those who were not lost to follow-up/withdrawal from the study by treatment arm........ 49

Table SM7. Number (%) young infants followed on day 2, 4, 8 and 15 by treatment arm........ 50

TABLE SM1: CLASSIFICATION AND MANAGEMENT OF YOUNG INFANTS WITH ANY SIGN OF POSSIBLE SERIOUS BACTERIAL INFECTION (PSBI)[1]

Clinical signs	Classification	Treatment or referral
- Convulsions or<br>- Not able to feed at all or, not feeding well, or<br>- Movement only when stimulated or no movement at all or<br>- High body temperature (≥38°C*) or<br>- Low body temperature (<35·5°C*) or<br>- Severe chest indrawing or<br>- Fast breathing (60 breaths per minute or more) in infants less than 7 days old	Possible serious bacterial infection (PSBI)	- Give the first dose of intramuscular antibiotics<br>- Refer URGENTLY to a hospital
If referral is not feasible, sub-classify as:		
**Clinical signs**	**Sub-classification**	**Treatment or referral**
- Convulsions or<br>- Not able to feed at all or<br>- No movement on stimulation or	Critical Illness	- Reinforce URGENT referral<br>- If the referral is still not feasible, give once-daily intramuscular gentamicin and twice-daily intramuscular ampicillin until a referral is feasible or for 7 days.
- Not feeding well on observation or<br>- Temperature 38°C* or more†<br>- Temperature less than 35·5°C* or<br>- Severe chest indrawing† or<br>- Movement only when stimulated	Clinical Severe Infection (CSI)	- Give once-daily intramuscular gentamicin and oral amoxicillin for 7 days.
- Fast breathing (60 breaths per minute or more) in infants less than 7 days old†	Severe Pneumonia	- Give oral amoxicillin for 7 days.

*Threshold based on axillary temperature

† These signs were identified as a low-mortality risk sign of PSBI[2]

References

1. World Health Organization. Integrated Management of Childhood Illness: management of the sick young infant aged up to 2 months. IMCI chart booklet. Geneva, Switzerland: WHO, 2019 (Available at: https://www.who.int/publications/i/item/9789241516365).

2. Nisar YB, Tshefu A, Longombe AL, et al. Clinical signs of possible serious infection and associated mortality among young infants presenting at first-level health facilities. *PLOS ONE* 2021; **16**(6): e0253110.

TABLE SM2: ETHICS APPROVALS, PARTICIPATING HOSPITALS, DATES FOR THE INITIATION AND END OF ENROLMENT BY STUDY SITE

Sites	Approval Bodies	Name of the Study Hospitals	Initiation and End Date of Enrolment
**The World Health Organization**	Ethics Review Committee for all research sites (ERC.0003289)	Not applicable	
**Bangladesh**	• National Research Ethics Committee, Bangladesh Medical Research Council for all sites in Bangladesh (27406022020)<br>• Institutional Review Board, Johns Hopkins Bloomberg School of Public Health, Baltimore, MD, USA (11306)	1. Zakiganj Upazila Health Complex, Sylhet<br>2. Sunamganj 250 Bed District Sadar Hospital, Sylhet<br>3. Moulvibazar 250 Bed District Sadar Hospital, Sylhet<br>**4.** 4. Habiganj District Hospital, Sylhet	June 26, 2021 to April 10, 2024
**Ethiopia**	• National Research Ethics Committee, Ministry of Education for all sites in Ethiopia (RE/141/9148/21)<br>• Addis Ababa University, College of Health Sciences Institutional Review Board for all sites in Ethiopia (050/20/SPH)	1. Adama Hospital Medical College<br>2. Bishoftu General Hospital<br>3. Batu General Hospital<br>4. Asella Teaching and Referral Hospital<br>5. Tirunesh Beijing General Hospital	Feb 2, 2022 to April 26, 2024
**India (Kanpur and Agra Sites, Uttar Pradesh)**	• Health Ministry's Screening Committee (HMSC), Department of Health Research (DHR) of the Indian Council of Medical Research (ICMR), New Delhi for all research sites in Uttar Pradesh (2020-10119)<br>• Community Empowerment Lab (CEL) Institutional Ethics Committee for all sites in Uttar Pradesh (CEL/RES/202007/001)	1. Hallet Hospital, Kanpur Nagar<br>2. Dufferin Hospital, Kanpur Nagar<br>3. Shyam Children Charitable Hospital, Kanpur Nagar<br>4. District Women Hospital, Kanpur Dehat<br>5. Sarojini Naidu Medical College, Agra<br>6. District Women Hospital, Agra	August 7, 2021 to April 26, 2024

	• Ethics Committee, to Ganesh Shankar Vidyarthi Memorial (GSVM) Medical College, Kanpur (CE/104/July/2020)<br>• Institutional Ethics Committee, Sarojini Naidu Medical College, Agra (SNMC/IEC/2022/65)		
**India (Himachal Pradesh and National Capital Region (NCR)**	• Health Ministry's Screening Committee (HMSC), Department of Health Research (DHR) of the Indian Council of Medical Research (ICMR), New Delhi for all research sites (2021- 0047/F1)<br>• Ethics Review Committee, Society for Applied Studies, New Delhi, for all research sites (SAS/ERC/PSBI-RCT-Study-1/2020)<br>• Institutional Ethics Committee, Dr YS Parmar, Government Medical College, Nahan, Himachal Pradesh (HFW/ME/DYSPGMC/IEC/2020/06)<br>• Institutional Ethics Committee, Indira Gandhi Medical College and Hospital, Shimla, Himachal Pradesh (HFW(MC-II) 13 (12)ETHICS/2020-15672)<br>• Biomedical Research Ethics Committee, Pt. BD Sharma PGIMS/UHS, Rohtak, Haryana (BREC/22/40)<br>• Institutional Ethics Committee, Dr Baba Saheb Ambedkar Medical College and Hospital, Delhi (5	1. Dr. Yashwant Singh Parmar, Government Medical College, Nahan, Himachal Pradesh<br>2. Civil Hospital, Paonta Sahib, Himachal Pradesh<br>3. Indira Gandhi Medical College and Hospital, Shimla, Himachal Pradesh<br>4. Pt. Bhagwat Dayal Sharma, Post Graduate Institute of Medical Sciences, Rohtak, Haryana<br>5. Dr Baba Saheb Ambedkar Medical College and Hospital, Rohini, Delhi<br>6. Lady Hardinge Medical College and Associated Hospitals, New Delhi	September 1, 2021 to April 15, 2024

	• (32)2020/BSAH)DNB/PF/22594-95)<br>• Institutional Ethics Committee, Lady Hardinge Medical College and Associated Hospitals, New Delhi (LHMC/IEC/2023/04)		
**Nigeria**	• Ahmadu Bello University Teaching Hospital Health Research Ethics Committee (ABUTHZ/HREC/W32/2020)	1. Hajiya Gambo Sawaba General Hospital, Zaria.<br>2. General Hospital, Giwa<br>3. Yusuf Dantsoho Memorial Hospital, Tudun Wada Kaduna	June 24, 2021 to March 9, 2024
**Pakistan**	• National Bioethics Committee for all research sites (4-87/NBC-509/20/603)<br>• Institutional Review Board, The Aga University Ethics Review Committee for all research sites (2020-3594-8920)	1. The Aga Khan Hospital for Women and Children Kharadar (Jan Bai Hospital)<br>2. National Institute of Child Health (NICH)<br>3. Sindh Government Children Hospital (SGCH)<br>4. Sindh Institute of Child Health and Neonatology (SICHN)	September 7, 2021 to August 12, 2024
**Tanzania**	• Institutional Review Board: National Institute Of Medical Research for all research sites (NIMR/HQ/R.8a/Vol. IX/3492)<br>• Institutional Review Board: Muhimbili University of Health And Allied Sciences for all research sites (MUHAS-REC-04-2020-081)<br>• Institutional Review Board, Tanzania Medicine and Medical Devices (TMDA0020/CTR/0001/05)<br>• Institutional Review Board, Harvard School of Public Health, Harvard University, Boston, MA, United States (IRB20-0119)	1. Amana Regional Referral Hospital<br>2. Temeke Regional Referral Hospital	June 24, 2021 to December 11, 2023

Panel 1: Study eligibility criteria for enrolment

**Inclusion criteria**
- Age less than 2 months, and<br>- Living in an area where they could be followed for 15 days, and<br>- Presented with any one of the following single low mortality risk sign of possible serious bacterial infection (PSBI)<br>o Body (axillary) temperature ≥38°C<br>o Severe chest indrawing<br>o Fast breathing (≥60 breaths per minute in 0-6 day-old infants)
**Exclusion criteria**
- Any other sign of possible serious bacterial infection (PSBI) as given below:<br>o Stopped feeding well<br>o Movement only on stimulation<br>o Low body (axillary) temperature <35.5°C<br>o More than one sign of clinical severe infection (CSI)<br>o Convulsions<br>o Not able to feed at all<br>o No movement at all.<br>- Other exclusions<br>o Weight < 2 kg at the time of presentation (if age at screening is less than 10 days) or weight for age <-3z<br>o Any sign suggestive of another serious illness/condition, such as major congenital malformations, severe jaundice, conditions requiring major surgery, meningitis, bone or joint infection, severe dehydration, hypoxaemia (oxygen saturation <90%)*, etc.<br>o Hospitalised for any illness in the previous two weeks<br>o Prior use of injectable antibiotics for the same illness in the last two days, except as a pre-referral dose<br>o Appearance of low mortality risk sign within the first 24 hours of life<br>o Previously included in this study or currently included in any other study

*****Hypoxaemia was added as an exclusion criterion from September 2023 onwards, based on the recommendation of the Data Safety Monitoring Board (DSMB)

OPTIMISING PLACE OF TREATMENT FOR YOUNG INFANTS AGED LESS THAN TWO MONTHS WITH ANY LOW-MORTALITY-RISK SIGN OF POSSIBLE SERIOUS BACTERIAL INFECTION (PSBI)

STATISTICAL ANALYSIS PLAN FOR THE PRIMARY ANALYSIS

Universal Trial Number (UTN): U1111-1251-1576

Ethics approval from WHO ERC (ERC0003289) and the ethics committee at each study site.

**VERSION:**	Version 1.0
**DATE:**	09/28/2023 (Last update: 07/10/2024)
**SPONSOR:**	World Health Organization (WHO)
**FUNDER:**	This work was supported by Bill and Melinda Gates Foundation grant number INV001311
**PREPARED BY:**	WHO, PSBI Clinical Site Investigators and Research Triangle Institute (RTI) International
**PSBI STUDY**:	☒RCT1<br>☐RCT2<br>☐Critically Ill

TYPE OF DATASET: ☒Pooled data from all sites

☐Site-specific data. Specify site: ________________________

☐Region-specific data. Specify region: ___________________

Date of modifications and list of changes:

04/24/2024

- Limit population to those screened during the time of the study
- Added supplement tables by site

06/20/2024:

- Updated the ampicillin in-hospital full treatment to allow for a dose on Day 7. This is based on Pakistan's confirmation that the infant may only receive one dose if discharged in the morning on Day 7.

07/10/2024:

- Added a footnote of the ssummarised other reasons
- Changed "<7 days old" to "≥24 hours to <7 days old".
- Added a footnote for the study exclusion criteria for weight-for-age to explain how we have babies with WAZ < - 2.
- Added footnote to outcome tables about the proportion for the signs being out of the number with the outcome.
- Corrected #5 persistent sign in the outcome – the signs are mutually exclusive.
- Tanzania also confirmed that the infant may only receive one dose if discharged in the morning on Day 7. This definition has already been updated based on Pakistan's responses. So, there is no additional changes to the variables.

Contents

Section 1. Statement of Problem 11
1.1. Background and Rationale 11
1.2. Analysis Aims and Outcomes 12
1.2.1. Analysis Aims 12
1.2.2. Primary Outcomes 13
1.2.3. Secondary Outcomes 13
1.2.4. Safety Outcomes 13
1.3. Primary Hypothesis 14
1.4. Secondary Hypotheses 14

Section 2. Analysis Plan 14
2.1. Population of Interest 14
2.2. Treatment Arms and Randomisation 14
2.2.1. Intervention 14
2.2.2. Control 14
2.2.3. Randomisation 15
2.3. Analysis Populations 15
2.3.1. Intention-to-Treat (ITT) Population 15
2.3.2. Per-Protocol (PP) Population 15
2.3.3. Safety (SAF) Population 15
2.4. Sample Size 15
2.5. Statistical Analysis Plan 16
2.5.1. Consort 16
2.5.2. Baseline Characteristics 17
2.5.3. Aim 1: Primary Analysis 17
2.5.4. Aim 2: Secondary Analysis 18
2.5.5. Aim 3: Treatment compliance 18
2.5.6. Safety Analysis 19
2.6. Variables of Interest 19
2.6.1. Study Inclusion / Exclusion Variables 19
2.6.2. Primary Outcome Variable 20
2.6.3. Secondary Outcome Variables 21
2.6.4. Safety Outcome Variables 21
2.6.5. Independent Variables 21

Section 3. Draft Tables and Figures 24
3.1. Draft Tables 24
3.2. Draft Figures 35

Section 4. Timeline.. 43

Section 5. References 44

SECTION 1. STATEMENT OF PROBLEM

1.1. BACKGROUND AND RATIONALE

The background and rationale are provided from the RCT1 published protocol:

PSBI Study Group. Optimal place of treatment for young infants aged less than two months with any low-mortality-risk sign of possible serious bacterial infection: Study Protocol for a randomised controlled trial from low- and middle-income countries. J Glob Health 2023;13:04055.

Neonatal mortality has substantially reduced over the last few decades, but an estimated 2.4 million neonatal deaths still occur worldwide annually, accounting for 47% of under-five deaths [1]. Neonatal infections account for over 35% of all neonatal deaths in South Asia and sub-Saharan Africa [2]. The World Health Organization (WHO) Integrated Management of Childhood Illness (IMCI) algorithm classifies neonates and young infants with clinically suspected sepsis as "Possible Serious Bacterial Infection (PSBI)" [3]. This classification is based on seven clinical signs – fast breathing (≥60 breaths per minute) in 0-6 days old babies, severe chest indrawing, high body temperature (≥38°C), low body temperature (<35.5°C), not able to feed at all or not feeding well / stopped feeding well, convulsions, and movement only when stimulated or no movement at all [3]. The WHO guideline recommends that young infants with any sign of PSBI should be managed in a hospital with injectable antibiotics and supportive care [4]. When referral to a hospital is not feasible, the WHO guideline recommends further classification of these infants into those who are critically ill (**Box 1**) and those who have a clinical severe infection (CSI) [5]. CSI can be managed on an outpatient basis with injectable gentamicin for two or seven days and oral amoxicillin for seven days based on clinical trials from Africa [6,7] and Asia [8,9].

Several countries have now adopted the WHO PSBI management guideline [5]. Implementation research in several countries in Africa and Asia on the WHO PSBI guideline has demonstrated that this guideline can be scaled up and that outpatient treatment is safe and effective when hospitalisation is not feasible [10-22]. It is estimated that 8%-12% of all young infants had at least one episode of PSBI within the first two months of life. Overall, approximately a quarter to half of the sick young infants in different settings accept hospital referrals [6,8,23]. However, hospitalisation also has risks, particularly that of nosocomial infections, including those from multi-drug resistant pathogens [24-27]. Therefore, only those young infants with signs of PSBI who have a favourable benefit-risk ratio should be hospitalised. Secondary observational analyses of AFRIcan NEonatal Sepsis Trial (AFRINEST) [6,7] data showed that some of the more common clinical signs of PSBI are associated with relatively low mortality [28].

Box 1

Sub-classification of children with signs of possible serious bacterial infection when a referral is not feasible [5].

Critical illness

- convulsions
- not able to feed at all
- no movement at all

Clinical Severe Infection

Low-mortality risk signs

- high body temperature (≥38°C*)
- severe chest indrawing
- fast breathing of ≥60 breaths per minute in ≥ 24 hours to < 7 days old infants

Specifically, having only fever (temperature ≥38°C) in infants 0-59 days of age, only severe chest indrawing in infants 0-59 days of age, or only fast breathing in infants 0-6 days of age had relatively low case fatality risk (CFR) of 0.8%, 0.9% and 2.0%, respectively [28] as compared to movement only on stimulation (CFR = 4%) or low body temperature (CFR = 11.0%) which had higher mortality. Infants presenting with multiple signs of CSI (two or more) also had a moderate risk of mortality (CFR = 5.7%). As expected, signs of critical illness were associated with a very high risk of death (convulsions, CFR = 11.3%, unable to feed at all, CFR = 22.9% and no movements at all, CFR = 25.0%).

An important implication of these findings is that young infants with signs associated with a relatively lower risk of mortality (fast breathing with ≥60 breaths per minute in 0-6 days of age, or temperature ≥38°C or severe chest indrawing in 0-59 days of age) may not require a referral for inpatient treatment in a hospital for one week per current WHO guidelines [4]. If infants with low-risk signs of PSBI could be managed at the outpatient level, it may reduce the referral for hospitalisation by over 70%. On the other hand, infants who have the other signs of CSI (stop feeding well, movements only on stimulation, low body temperature), with multiple signs of CSI, as well as those who have a critical illness, are at a high risk of mortality [28] and may have a more favourable benefit-risk ratio for hospitalisation.

When mortality data from AFRINEST were analysed by place of treatment for all infants classified as PSBI, CFR was higher in hospitalised young infants as compared to infants treated on an outpatient basis when they refused referral for the same signs of CSI [28]. The overall CFR for young infants with CSI treated at the hospital was three times higher (6.5%) compared to those treated on an outpatient basis (1.9%) [28]. Nevertheless, due to the observational nature of the AFRINEST study and specifically that infants who refuse referral may be systematically different from those who accept, the findings cannot be considered causal. Therefore, there was a need to evaluate this in a randomised controlled trial.

1.2. ANALYSIS AIMS AND OUTCOMES

1.2.1. Analysis Aims

The study has the following aims:

Aim 1

The primary objective of this trial is to measure the effect of outpatient treatment on the composite poor clinical outcome compared with inpatient treatment in young infants 0-59 days old with only one low-mortality risk sign of CSI. Specifically, among young infants < 2 months old with only one low-mortality risk CSI sign (high body temperature ≥38ºC, severe chest indrawing, fast breathing of ≥ 60 breaths per minutes in 1-6 days old infants) presenting to outpatient/emergency department of a hospital (**Population**), does outpatient treatment with injectable gentamicin for 2 days and oral amoxicillin for 7 days **(Intervention)**, compared to the currently recommended inpatient hospital treatment initiated with injectable ampicillin and gentamicin and supportive care (**Control**), result in lower rates of poor clinical outcome (**Outcome**)?

Aim 2

A secondary objective of this trial is to measure the effect of outpatient treatment on the individual components of poor clinical outcome compared with inpatient treatment in young infants 0-59 days old with only one low-mortality risk sign of CSI. Specifically, the individual components of the poor clinical outcome are: 1) death, 2) critical illness, 3) another serious infection, 4) any new CSI and 5) persistence of the presenting CSI sign.

Aim 3

Assess treatment compliance/medication adherence by treatment group.

1.2.2. Primary Outcomes

The primary outcome of the study is a poor clinical outcome, which is defined as any of the following: 1) death at any time from randomisation up to day 15 of initiation of therapy, or 2) any sign of critical illness (no movement at all, unable to feed at all, or convulsions) on day 2, 4 or day 8 post-randomisation, or 3) any sign suggestive of another serious infection, eg, meningitis, bone or joint infection on day 2, 4 or day 8 post-randomisation, or 4) any new CSI sign on day 4 or day 8 post-randomisation, or 5) persistence of the presenting CSI sign on day 8 post-randomisation.

1.2.3. Secondary Outcomes

Secondary outcomes include the individual components of poor clinical outcome, specifically: 1) death, 2) critical illness, 3) another serious infection, 4) any new CSI and 5) persistence of the presenting CSI sign.

1.2.4. Safety Outcomes

Any adverse event that occurs after enrolment was recorded on an adverse event reporting form by the treating nurse / physician. In case of an SAE, the study staff contacted the study supervisor and IOA. The IOA documented the SAE and conveyed the information to the study coordinator / investigator. An SAE, like death, anaphylactic reaction, severe diarrhoea, and a disseminated or severe rash, was reported to WHO within 48 hours of the occurrence. These cases were (except the ones who unfortunately die) referred for appropriate treatment and followed up. In case of other minor adverse effects, such as mild rash etc., the treatment was continued. WHO reported SAEs to the Data Safety Monitoring Board (DSMB). The DSMB was an independent group who monitored the trial at regular intervals.

The safety outcomes reviewed by the DSMB on a 3-month schedule were:

- Study deaths
- Any other serious adverse event and whether the event is related to treatment. These cases will be considered treatment failure.
    - anaphylactic reaction,
    - other likely allergic reaction including disseminated rash,
    - infection or abscess at injection site,
    - diarrhoea with severe dehydration,
    - other adverse event.
- Critical illness
    - no movement at all,
    - unable to feed at all,
    - convulsions

Study deaths and critical illness are already covered under the primary outcome of poor clinical outcome.

1.3. PRIMARY HYPOTHESIS

The primary hypothesis is that young infants with only one low-mortality risk sign of CSI presenting to outpatient/emergency department of a hospital, who receive outpatient treatment, will experience a better (s*uperiority hypothesis*), or at least non-inferior (*non-inferiority hypothesis*), clinical outcome than young infants that receive inpatient treatment when comparing the composite variable of poor clinical outcome in the groups.

1.4. SECONDARY HYPOTHESES

The secondary hypothesis is that young infants with only one low-mortality risk sign of CSI presenting to outpatient/emergency department of a hospital, who receive outpatient treatment, will experience a better (s*uperiority hypothesis*), or at least non-inferior (*non-inferiority hypothesis*), clinical outcome than young infants that receive inpatient treatment when comparing the individual components of poor clinical outcome in the groups.

SECTION 2. ANALYSIS PLAN

2.1. POPULATION OF INTEREST

Participants who are eligible and consenting to:

☒RCT1

☐RCT2

And/or were followed-up in the critically ill group:

☐Critically Ill

2.2. TREATMENT ARMS AND RANDOMISATION

2.2.1. Intervention

Outpatient treatment with injectable gentamicin (once daily) for two days plus oral amoxicillin (twice daily) for seven days.

2.2.2. Control

Inpatient antibiotic treatment initiated with WHO recommended antibiotic regimen – injectable ampicillin (2+ times a day) plus injectable gentamicin (once daily) along with other supportive care for 7-10 days.

2.2.3. Randomisation

Participants were individually allocated to inpatient or outpatient treatment with a block randomisation scheme. WHO staff in Geneva not associated with site work independently prepared the randomisation list in block sizes of 2, 4, 6 and 8 and shared it with the Research Triangle Institute (RTI), the study Data Coordination Centre (DCC). Allocation concealment was ensured using QR randomisation codes. Randomisation was in a 1:1 ratio. RTI produced a list of encrypted QR codes and had these printed on labels based on the randomisation scheme for each site and study. RTI transmitted the printed QR code with blue colour labels to the sites. There were three labels per infant: one each for the log register, patient card, and hospital file or outpatient department (OPD) slip.

Each hospital within the site had a single set of QR codes that all screening and enrolment team members used, even if the screening and enrolment took place in different locations within the hospital. After determining eligibility, the data collector took the QR code label next in the numeric sequence from the list at their facility and scanned this to obtain the randomisation number.

2.3. ANALYSIS POPULATIONS

2.3.1. Intention-to-Treat (ITT) Population

For an intention-to-treat (ITT) analysis all infants who are randomised will be included in the statistical analysis. ITT is more conservative analysis with slightly wider confidence intervals if there is a difference by allocation group. Additionally, our study outcome could occur any time between date of enrolment till day 15. Of note, in general, the variables for the PSBI ITT analyses are coded as "Yes" when the condition is present, "No" if information is available at any visit and the outcome is not present, and "LFU/withdrawn consent" if there is no outcome information available for the case and the child is either lost-to-follow-up or consent was withdrawn. However, parents / caregivers could withdraw at any time after enrolment, and those who withdraw at any stage from the study continued to receive free-of-charge standard treatment and treating physicians followed them as per hospital standard procedures. These cases are included in the intention-to-treat analysis unless they refused to be part of the analyses.

2.3.2. Per-Protocol (PP) Population

The per protocol analysis population is the same as the intention-to-treat analysis population with three additional exclusions. Those who are "LFU/withdrawn consent" will not be included in the per-protocol analysis. Additionally, any ineligible infants whose family consented to the study and were enrolled will be excluded. Infants who did not initiate the study medication will also be excluded.

2.3.3. Safety (SAF) Population

The safety population includes infants who have outcome information on day 2, day 4 or day 8 or infant died or had another serious adverse event at any time after randomisation.

2.4. SAMPLE SIZE

Sample size was calculated to be able to test both the superiority and non-inferiority hypotheses.

1. ***Intervention is superior to standard of care in reducing poor clinical outcomes:*** Assuming that 6% of infants in the standard care group will have poor clinical outcome (as defined above,

based on AFRINEST study data), and 95% confidence level and 90% power, we will need 3135 infants per group for detecting a 30% lower outcome in the intervention group (4.2% vs 6.0%). We will be able to detect a 25% lower outcome in the intervention group (4.5% vs. 6.0%) with 80% power if we enrol 3468 infants per group.

2. ***Intervention is non-inferior to standard of care with respect to poor clinical outcomes:*** Assuming that 6% of infants in both the intervention and standard care group will have a poor clinical outcome, 95% confidence level (one-sided), 90% power and a 1.8% non-inferiority margin, we will need to enrol 2983 infants per group. With 80% power and a 1.5% non-inferiority margin, we will need to enrol 3101 infants per group.

We will therefore enrol a total of 7000 infants with a single low-mortality risk sign in Study 1. The Study Data Safety Monitoring Board (DSMB) will perform the interim analyses at 25%, 50% and 75% of enrolment and if required, will recalculate the sample size to enable us to answer the research question.

This sample size will allow us to meet the superiority and inferiority targets given in Table 1.

Table 1. Sample size calculation for the trial design

Trial design	% poor clinical outcome in the control arm [6,7]	% poor clinical outcome in the interventional arm	Power	% lower outcome or noninferiority margin	Sample size needed
Superiority	6.0	4.0	90%	30	6270
Superiority	6.0	4.5	80%	25	6938
Noninferiority	6.0	6.0	90%	1.8	5966
Noninferiority	6.0	6.0	80%	1.5	6202

2.5. STATISTICAL ANALYSIS PLAN

The study took place in seven clinical sites across six countries. The clinical sites are Bangladesh, Ethiopia, India HP, India UP, Nigeria, Pakistan and Tanzania.

We will look at the relationships overall and by region due to large differences between regions for the risk factors and outcomes. Sites will be grouped into two regions as follows:

- Africa: Ethiopia, Nigeria, and Tanzania
- Asia: Bangladesh, India HP, India UP, and Pakistan

For the modeling of the poor clinical outcomes, site will be included in a sensitivity analysis, as the randomisation was done by site. Additionally, as explained above, the targeted enrolment by site was 1,000 infants. However, due to a number of recruitment challenges, the scientific advisory board approved the sites continuing enrolment after completing these targets in order for the study to meet the overall enrolment goal of 7,000 infants. This change resulted in some sites enrolling more participants than others.

2.5.1. Consort

Provide consort information from screening through randomisation among those who consented to screening with at least one sign of PSBI during the RCT1 study period.

- Table 1 – Consort table overall and by region
- Figure 1 – Consort diagram (all sites combined)
- Supplement Table 1 – Consort table overall and by site

2.5.2. Baseline Characteristics

Provide means and standard deviations as well as proportions of baseline characteristics and presenting risk signs of PSBI.

- Table 2. Infant baseline characteristics and presenting PSBI risk signs by treatment group (inpatient and outpatient): Overall and stratified by region.
- Supplement Table 2. Infant baseline characteristics and presenting PSBI risk signs by treatment group (inpatient and outpatient): Overall and stratified by site.

2.5.3. Aim 1: Primary Analysis

The analysis of Aim 1 will be completed for both the intention-to-treat (for testing the superiority hypothesis) and per-protocol (for testing the non-inferiority hypothesis) populations. *(See above for a description of the analysis populations.)* The primary outcome rates of poor clinical outcome will be compared between the intervention and control groups overall and by region. Of note, this is a participant-based analysis in which one infant is counted for one outcome in the hierarchical order above. For example, if the infant has both critical illness (#2 above) and another serious illness (#3 above), then this infant is only counted once in critical illness. Signs under each outcome are not mutually exclusive, meaning that one infant can have more than one sign within each outcome. For example, an infant with any new sign of CSI (#4 above) may have experienced both severe chest indrawing and stopped feeding well.

For the overall intent-to-treat population, the superiority of the study treatment is tested using a Farrington-Manning (1990) score test with a superiority margin of 30% which compares the favorable outcome (i.e. those with either no poor clinical outcome or those who were lost to follow-up/withdrew consent) in the outpatient treatment arm to the inpatient control arm. The resulting p-value from the test will be displayed. A significant p-value indicates superiority is met. Risk differences and 95% confidence intervals for poor clinical outcome will be displayed numerically and in forest plots. Additionally, a risk difference controlling for clinical site will be calculated among the intent-to-treat population as a sensitivity analysis. This analysis allows us to consider the effect of the clinical sites, as the randomisation was done by site and the total number of participants enrolled differs by site. The unadjusted and adjusted risk differences (and associated 95% CI) for poor clinical outcome will be obtained by fitting sgeneralised linear models to experiencing a poor clinical outcome with the following fixed effects: treatment group and site (adjusted analysis only).

For the per-protocol analysis, the non-inferiority of the study treatment is tested using a Farrington-Manning (1990) score test with a non-inferiority margin of 1.8% which compares the favorable outcome (i.e. those with no poor clinical outcome) in the outpatient treatment arm to the inpatient control arm. The resulting p-value from the test will be displayed. A significant p-value indicates non-inferiority is met. Risk differences and 95% confidence intervals for poor clinical outcome will be displayed numerically and in forest plots. Additionally, a risk difference controlling for clinical site will be calculated among the per-protocol population as a sensitivity analysis. This analysis allows us to consider the effect of the clinical sites, as the randomisation was done by site and the total number of participants enrolled differs by site. The unadjusted and adjusted risk differences (and associated 95% CI) for poor clinical outcome will be obtained by fitting sgeneralised linear models to experiencing a poor clinical outcome with the following fixed effects: treatment group and site (adjusted analysis only).

No adjustment will be made for control of type I error due to two primary outcomes, because the two outcomes are complementary and therefore a correction is not warranted.

Descriptive statistics will be provided for regional data. However, no formal testing will be done for the individual regions.

- Figure 2. Superiority analysis of the primary and secondary outcomes and risk differences by treatment group among the overall intent-to-treat population.
- Figure 3. Non-inferiority analysis of the primary and secondary outcomes and risk differences by treatment group among the overall per-protocol population.
- Table 3. Poor clinical outcome rates by treatment group among the intent-to-treat population stratified by region.
- Table 4. Poor clinical outcome rates by treatment group among the per-protocol population stratified by region.
- Supplement Table 3. Poor clinical outcome rates by treatment group among the intent-to-treat population stratified by site.
- Supplement Table 4. Poor clinical outcome rates by treatment group among the per-protocol population stratified by site.

2.5.4. Aim 2: Secondary Analysis

The individual components of poor clinical outcome will be analysed along with the primary outcome following the same approach as outlined above. Specifically, the individual components of the primary outcome are: 1) death, 2) critical illness, 3) another serious infection, 4) any new CSI and 5) persistence of the presenting CSI sign. Of note, this is a participant-based analysis in which one infant is counted for one outcome in the hierarchical order above. For example, if the infant has both critical illness (#2 above) and another serious illness (#3 above), then this infant is only counted once in critical illness. Signs under each outcome are not mutually exclusive, meaning that one infant can have more than one sign within each outcome. For example, an infant with any new CSI (#4 above) may have experienced both severe chest indrawing and stopped feeding well.

These results will be included in Figures 2-3, Tables 3-4 and Supplement Tables 3-4 above.

2.5.5. Aim 3: Treatment compliance

The percent compliant to the treatment (defined as the location of the infant during the trial, the initiation of medications, taking at least 80% of the medications, and taking all of the medications, as applicable) will be calculated by treatment group for the overall population and by region. Table 5 is provided for reference. The applicable percentages will be reported in the text.

- Table 5. Summary of the location of the infant during the trial and receipt of study medication by treatment group overall and stratified by region.
- Supplement Table 5. Summary of the location of the infant during the trial and receipt of study medication by treatment group overall and stratified by site.

2.5.6. Safety Analysis

The number and percent of any SAE and type of SAE will be provided by treatment group overall and stratified by region. Since death is part of the poor clinical outcome. The number and percent of SAEs excluding death will be provided. Critical illness was also reported to the DSMB as a safety outcome. However, since this is included in poor clinical outcome, it will not be included here.

- Table 6. Severe adverse events (excluding deaths) and treatment failure by treatment group overall and stratified by region.
- Supplement Table 6. Severe adverse events (excluding deaths) and treatment failure by treatment group overall and stratified by site.

2.6. VARIABLES OF INTEREST

Please refer to the document **PSBI Variable Documentation.docx** for detailed information on how each of the variables below were constructed. The documentation includes the brief variable description, forms the variable is collected on, notes on how the variable was derived (if applicable), variable name and codes.

2.6.1. Study Inclusion / Exclusion Variables

Limit screening dataset to infants with 1 or more sign of PSBI who consented to screening. Also, exclude screening records that happened after the screening for RCT1 was completed for the sites. RCT1 stop date cut offs for a1_screening_date by site are as follows:

- Bangladesh (sitenum=1) 09Mar24
- Ethiopia (sitenum=2) 20Apr24
- India HP (sitenum=3) 13Apr24
- India UP(sitenum=4) 20Apr24
- Nigeria (sitenum=5) 07Mar24
- Pakistan(sitenum=6) 08Mar24
- Tanzania(sitenum=7) 11Dec23

Study inclusion/exclusion variables are:

- Single mortality risk sign (single_low_mort_no_worse=1)
- Inclusion met (RCT1_inclusion_met=1)
    - Lives in geographic area where FUP can be completed (a1_geo_area_follow_12=1)
- Exclusion met (RCT1_exclusion_met=1)
    - Underweight (a1_underweight_12=1)
    - Serious illness or condition – this is a summary category that includes any of the following:
        - Another serious illness/condition (major congenital malformation (a1_infant_signs_malf_found_12=1), severe jaundice (a1_infant_signs_jaund_found_12=1), condition requiring major surgery (a1_infant_signs_surg_found_12=1), meningitis (a1_infant_signs_menin_found_12=1), bone or joint infection (a1_infant_signs_infect_found_12=1), severe dehydration (a1_infant_signs_dehyd_found_12=1), , other serious illness(a1_othr_ill_yn_found_12=1))

- Hypoxaemia (a1_infant_hypoxaemia_found_12=1)
- <24 hours old when PSBI signs presented – this is a summary category that includes any of the following:
  - < 24 hours old (a1_age24hrs_12=1)
  - Fast or difficult breathing or fever in first 24 hours of life (a1_first_24hr_breathing_12=1)
- Already receiving care before screening – this is a summary category that includes any of the following:
  - sHospitalised in previous 2 weeks (a1_hosp_twoweeks_12=1)
  - Received injectable antibiotics for same illness in last 2 days (a1_inject_antibiotics_12=1)
- Enrolled previously in same study or currently enrolled in another study
  - Enrolled previously in same study (a1_prev_enroll_12=1)
  - Currently included in other study (a1_any_study_12=1)

- Other exclusion
  - Sample size reached, participant not consented (RCT1_samp_reached=1)
- Eligible (RCT1_eligibility_met=1)
- Consent (a1_consent_given=1)
- Enrolled (RCT1_enrolled=1)
- Randomised (RCT1_trt=1 or 2) – the intent-to-treat population includes all randomised subjects

Additional inclusion/exclusion variables for this analysis:

- Per-protocol population (RCT1_perprotocol_pop=1) excludes the following:
  - Those who are "LFU/withdrawn consent" and the poor clinical outcome could not be calculated (rct1_poor_clinical_outcome_DSMB=3)
  - Ineligible infants whose family consented to the study and were enrolled ((RCT1_trt=1 or 2, but RCT1_eligibility_met not equal 1).
  - Infants who did not initiate the study medication (Control inpatient group and did not start study medications (a2_inpatient_start_trt not equal 1) or Outpatient treatment group and did not start study medications (a2_outpatient_start_trt not equal 1)).

2.6.2. Primary Outcome Variable

The primary outcome variable for poor clinical outcome is rct1_poor_clinical_outcome_DSMB, which has codes 1=Yes, 2=No, 3=LFU/withdrew. This is a complex composite variable which is coded, as follows:

Among those randomised and at least 15 days out from screening, poor clinical outcome defined as:

- Death any time from randomisation up to day 15 of initiation of therapy (c1_death_exp_DSMB=1), or
- Presence of any sign of critical illness (no movement at all, unable to feed at all, or convulsions) on day 2, 4 or day 8 of initiation of therapy (rct1_outcome_critical_ill_DSMB=1), or
- Any sign suggestive of another serious infection, e.g. meningitis, bone or joint infection, on day 2, 4 or day 8 of initiation of therapy (rct1_another_infection_DSMB=1), or

- Presence of any new sign of CSI on day 4 or day 8 of initiation of therapy (rct1_new_csi_DSMB=1), or
- Persistence of the presenting sign on day 8 of initiation of therapy (rct1_persistent_csi_d8_DSMB=1).

If any of the above are true then rct1_poor_clinical_outcome_DSMB=1.

Otherwise, if the participant had information gathered at visit 2, 4, or 8 (i.e. if critical illness is not missing (a3_critically_ill_d2, a3_critically_ill_d4, or a3_critically_ill_d8 not missing), or other serious infection is not missing (a3_suggest_infect_d2, a3_suggest_infect_d4, or a3_suggest_infect_d8), or information on CSI not missing at Day 4 or Day (a3_critically_ill_d4, a3_suggest_infect_d4, a3_chest_indrawing_d4, a3_high_body_temp_d4, a3_breath_count_1_d4, a3_breath_count_2_d4, a3_diff_feed_d4, a3_infant_mov_spont_d4, a3_mov_only_stim_d4, a3_low_body_temp_d4, a3_critically_ill_d8, a3_suggest_infect_d8, a3_chest_indrawing_d8, a3_high_body_temp_d8, a3_breath_count_1_d8, a3_breath_count_2_d8, a3_diff_feed_d8, a3_infant_mov_spont_d8, a3_mov_only_stim_d8, or a3_low_body_temp_d8 not missing) or the baby had an adverse event form (form_C1_1)) then rct1_poor_clinical_outcome_DSMB=2.

Otherwise, the infant is considered LTF/withdrawn then rct1_poor_clinical_outcome_DSMB=3.

2.6.3. Secondary Outcome Variables

The secondary outcome variables are the components that make up the poor clinical outcome. They have codes 1=Yes, 2=No, 3=LFU/withdrew and are as follows:

- Death any time from randomisation up to day 15 of initiation of therapy (c1_death_exp_DSMB=1),
- Presence of any sign of critical illness (no movement at all, unable to feed at all, or convulsions) on day 2, 4 or day 8 of initiation of therapy (rct1_outcome_critical_ill_DSMB=1),
- Any sign suggestive of another serious infection, e.g. meningitis, bone or joint infection, on day 2, 4 or day 8 of initiation of therapy (rct1_another_infection_DSMB=1), or
- Presence of any new sign of CSI on day 4 or day 8 of initiation of therapy (rct1_new_csi_DSMB=1),
- Persistence of the presenting sign on day 8 of initiation of therapy (rct1_persistent_csi_d8_DSMB=1).

2.6.4. Safety Outcome Variables

Safety outcomes include death (rct1_poor_clinical_death_DSMB=1), critical illness (rct1_poor_clinical_crit_ill_DSMB=1), or other serious infection (rct1_poor_clinical_oth_inf_DSMB=1). Additionally, treatment failure (rct1_outcome_trt_fail_DSMB=1), is also included.

2.6.5. Independent Variables

Independent variables (e.g., covariates/risk factors) include the following:

- Treatment group (RCT1_trt)
    - 1=Inpatient
    - 2=Outpatient

- Infant location per treatment group
  - Control inpatient treatment group in the hospital for Days 1-7 (a2_hosp_all_a)
    - 1=Yes
    - 2=No
  - Intervention outpatient treatment group at home or at a medical facility only to receive medication for Days 1-7 (a2_home_all_b)
    - 1=Yes
    - 2=No
- Initiated and received medication per treatment group
  - Control inpatient treatment group
    - Initiated medication (a2_inpatient_start_trt: 1=Yes, 2=No) is defined as:
      - at least one dose of injectable ampicillin on Day 1, and
      - at least one dose of injectable gentamicin on Day 1
    - Received at least 80% of medication (a2_inpatient_80_trt_a: 1=Yes, 2=No) is defined as:
      - at least 5 days of injectable ampicillin,
      - at least 5 doses of injectable gentamicin,
      - no other antibiotics for seven days
    - Received all medication (a2_inpatient_full_trt: 1=Yes, 2=No) is defined as:
      - at least one dose of injectable ampicillin on Day 1,
      - at least 2 doses of injectable ampicillin on Days 2-6,
      - at least one dose of injectable ampicillin on Day 7,
      - at least one dose of injectable gentamicin for seven days,
      - no other antibiotics for seven days
  - Intervention outpatient treatment group
    - Initiated medication (a2_outpatient_start_trt: 1=Yes, 2=No) is defined as:
      - at least one dose of injectable gentamicin on Day 1, and
      - at least one dose of oral amoxicillin on Day 1
    - Received at least 80% medication (a2_outpatient_80_trt: 1=Yes, 2=No) is defined as:
      - at least one dose of injectable gentamicin for two days,
      - at least ten doses of oral amoxicillin and
      - no other antibiotics for seven days
    - Received all medication (a2_outpatient_full_trt: 1=Yes, 2=No) is defined as:
      - at least one dose of injectable gentamicin for two days,
      - at least one dose of oral amoxicillin on Day 1,
      - at least two doses of oral amoxicillin on Days 2-7 and
      - no other antibiotics for seven days

- Site
  - 1=Bangladesh
  - 2=Ethiopia
  - 3=India HP
  - 4=India UP
  - 5=Nigeria
  - 6=Pakistan

    - 7=Tanzania
- Region (region)
    - 1=Africa (clinical sites Ethiopia, Nigeria, Tanzania combined)
    - 2=Asia (clinical sites Bangladesh, India HP, India UP, Pakistan combined)
- Age in days (infant_age) - Continuous
- Age less scategorised (infant_age_cat)
    - 1=≥ 24 hours to less than 7 days
    - 2=7-28 days
    - 3=29-59 days
- Infant sex (a1_sex)
    - 1=Male
    - 2=Female
    - 3=Undetermined
- Weight in grams (a1_weight_gms) - Continuous
- Weight-for-age z-score (_zwei) – Continuous
- Weight-for-age z-score categorized (_zwei_cat)
    - 1= < -2
    - 2= -2 to <-1
    - 3=-1 to 0
    - 4=> 0
- Presenting clinical signs
    - Severe chest indrawing (a1_chest_indrawing_12=1)
    - High body temperature (a1_high_body_temp_12=1)
    - Fast breathing ≥ 24 hours to < 7 days old (a1_fast_breath_signs_12=1)

SECTION 3. DRAFT TABLES AND FIGURES

3.1. DRAFT TABLES

Table 1: Consort overall and stratified by region

Variable name and value		Overall	Africa	Asia
No_sign_PSBI=2	1 or more signs of PSBI among young infants <2 months old who consented to screening, N			
single_low_mort_no_worse=1	Single low mortality risk sign, n(%)			
rct1_any_exclusion=1	Excluded, n (% of those with single low mortality risk sign)			
	Mutually exclusive exclusions, n (% of those excluded)			
rct1_any_exclusion_cat=1	Does not live in a geographic area where FUP can be completed			
rct1_any_exclusion_cat=2	Underweight			
rct1_any_exclusion_cat=3	Another serious illness or condition			
rct1_any_exclusion_cat=4	Hypoxaemia			
rct1_any_exclusion_cat=5	< 24 hours old when PSBI signs presented			
rct1_any_exclusion_cat=6	Already receiving care before screening.			
rct1_any_exclusion_cat=7	Enrolled previously in the same study or currently included in another study			
rct1_any_exclusion_cat=8	Did not consent			
rct1_any_exclusion_cat=9	Not randomised ← Replaced with "Other" and provide footnote of reasons consented, but not randomised.			
RCT1_ITT_pop=1	Randomised, n (% of those with single low mortality risk sign)			
	**Intent-to-treat population, n(%)**			
rct1_trt_itt=1	**Outpatient treatment arm**			
rct1_trt_itt=2	**Inpatient control arm**			
RCT1_perprotocol_pop=2	Excluded from the per-protocol population, n (% of the intent-to-treat population)			
	Mutually exclusive exclusions from per-protocol, n (% of those excluded)			
RCT1_pp_exclusion_cat=1	Lost-to-follow-up / withdrew consent			
RCT1_pp_exclusion_cat=2	Ineligible infants who were randomised			
RCT1_pp_exclusion_cat=3	Infants who did not initiate the study medication			

Variable name and value		**Overall**	**Africa**	**Asia**
	**Per-protocol population, n(%)**			
rct1_trt_pp=1	**Outpatient treatment arm**			
rct1_trt_pp=2	**Inpatient control arm**			

Table 2: Infant baseline characteristics and presenting risk signs by treatment group overall and stratified by region

Variable name and value: Treatment variable rct1_trt_itt		Overall			Africa			Asia		
		Total	Outpatient	Inpatient	Total	Outpatient	Inpatient	Total	Outpatient	Inpatient
RCT1_enrolled	**Infants, N**									
infant_age	Age (days), n									
	Mean (sd)									
	Median (P25, P75)									
	Categorised age (days), n(%)									
infant_age_cat=1	≥ 24 hours to < 7 days old									
infant_age_cat=2	7-28 days old									
infant_age_cat=3	29-59 days old									
a1_sex=1	Male, n (%)									
a1_weight_gms	Weight (g), n									
	Mean (sd)									
	Median (P25, P75)									
_zwei	Weight-for-age (z-score), n									
	Mean (sd)									
	Median (P25, P75)									
	Categorized weight-for-age (z-score)*, n (%)									
_zwei_cat=1	< -2									
_zwei_cat=2	-2 to <-1									
_zwei_cat=3	-1 to 0									
_zwei_cat=4	> 0									

Variable name and value: Treatment variable rct1_trt_itt		Overall			Africa			Asia		
		Total	Outpatient	Inpatient	Total	Outpatient	Inpatient	Total	Outpatient	Inpatient
	Risk signs among those enrolled									
a1_chest_indrawing_12	Severe chest indrawing, n(%)									
a1_high_body_temp_12	High body temperature (≥ 38C), n(%)									
a1_fast_breath_signs_12	Fast breathing ≥ 24 hours to < 7 days old, n(%)									

*Study exclusion criteria for weight-for-age was 'severely underweight' (cut off of < -3 WAZ) for infants >=10 days of age and for infants <10 days old it was weight <2000 grams.

Table 3. Poor clinical outcome rates by treatment group among the intent-to-treat population stratified by region.

Variable name and value:<br>Treatment variable rct1_trt_itt		Africa			Asia		
		Total	Outpatient	Inpatient	Total	Outpatient	Inpatient
RCT1_enrolled	**Enrolled, N**						
	Poor clinical outcome[1], n (%)						
rct1_poor_clinical_outcome_DSMB=1	Yes						
rct1_poor_clinical_outcome_DSMB=2	No						
rct1_poor_clinical_outcome_DSMB=3	LFU/withdrawn consent						
	Individual components of poor clinical outcome						
rct1_poor_clinical_death_DSMB=1	1. Death at any time from randomisation to day 15, n (%)*						
rct1_poor_clinical_crit_ill_DSMB=1	2. Critical illness day 2, 4 or 8, n (%)*<br>*signs not mutually exclusive*						
A3_infant_mov_none_d2_d4_d8_DSMB=1	*No movement at all*						
a3_unable_feed_d2_d4_d8_DSMB=1	*Unable to feed at all*						
A3_infant_fit_d2_d4_d8_DSMB=1	*Convulsions in the previous 24 hrs*						
rct1_poor_clinical_oth_inf_DSMB=1	3. Another serious illness at day 2, 4 or 8, n (%)*<br>*signs not mutually exclusive*						
A3_meningitis_d2_d4_d8_DSMB=1	*Meningitis*						
A3_bone_d2_d4_d8_DSMB=1	*Bone or joint infection*						
A3_infection_other_d2_d4_d8_DSMB=1	*Other serious infection*						
rct1_poor_clinical_new_csi_DSMB=1	4. Any new signs of CSI at day 4 or 8, n (%)*<br>*signs not mutually exclusive*						
a3_chest_indraw_new_d4_d8_DSMB=1	*Severe chest indrawing*						
a3_high_body_temp_new_d4_d8_DSMB=1	*High body temperature (≥ 38C)*						

Variable name and value: Treatment variable rct1_trt_itt		Africa			Asia		
		Total	Outpatient	Inpatient	Total	Outpatient	Inpatient
a3_fast_breath_new_d4_d8_DSMB=1	*Fast breathing ≥ 24 hours to < 7 days*						
a3_feed_diff_not_new_d4_d8_DSMB=1	*Stopped feeding well*						
a3_mov_only_stim_new_d4_d8_DSMB=1	*Movement only on stimulation*						
a3_low_body_temp_new_d4_d8_DSMB=1	*Low body temperature (<35.5C)*						
rct1_poor_clinical_pers_csi_DSMB=1	5. Persistence of the low mort risk sign at day 8, n (%)* *signs not mutually exclusive*						
a3_chest_indrawing_pers_d8_DSMB=1	*Severe chest indrawing*						
a3_high_body_temp_pers_d8_DSMB=1	*High body temperature (≥ 38C)*						
a3_fast_breath_pers_d8_DSMB=1	*Fast breathing ≥ 24 hours to < 7 days*						

[1]Poor clinical outcome defined as: Death between randomisation and day 15 of initiation of therapy, or presence of any sign of critical illness (no movement at all, unable to feed at all, or convulsions) on day 2, 4 or day 8 of initiation of therapy, or any sign suggestive of another serious infection, e.g. meningitis, bone or joint infection, on day 2, 4 or day 8 of initiation of therapy, or presence of any new sign (same sign as screening at day 8 after disappearing on day 4, or a different sign from screening on day 4 or 8) of CSI on day 4 or day 8 of initiation of therapy, or persistence of the presenting sign on day 8 (same sign at screening, day 4 and day 8) of initiation of therapy. Subset to those randomised and at least 15 days from screening. LTF/withdrawn consent and have no information gathered about the infants' outcome on Days 2, 4 or 8.

*This is a participant-based analysis in which one infant is counted for one outcome in the hierarchical order of the report from the reasons listed from 1. Death through 5. Persistent sign. For example, if the infant has both a critical illness and another serious illness, then this infant is only counted once in critical illness. The percentage provided is for the total intent-to-treat population.

NOTES: Timing of death is available from the completed SAE forms. Signs under each outcome are not mutually exclusive, meaning that one infant can have more than one sign within each outcome. The proportions for each sign are among those with the given outcome.

Table 4. Poor clinical outcome rates by treatment group among the per-protocol population stratified by region.

Variable name and value: Treatment variable rct1_trt_pp		Africa			Asia		
		Total	Outpatient	Inpatient	Total	Outpatient	Inpatient
RCT1_enrolled	**Enrolled, N**						
	Poor clinical outcome[1], n (%)						
rct1_poor_clinical_outcome_DSMB=1	Yes						
rct1_poor_clinical_outcome_DSMB=2	No						
	Individual components of poor clinical outcome						
rct1_poor_clinical_death_DSMB=1	1. Death at any time from randomisation to day 15, n (%)*						
rct1_poor_clinical_crit_ill_DSMB=1	2. Critical illness day 2, 4 or 8, n (%)*<br>*signs not mutually exclusive*						
A3_infant_mov_none_d2_d4_d8_DSMB=1	*No movement at all*						
a3_unable_feed_d2_d4_d8_DSMB=1	*Unable to feed at all*						
A3_infant_fit_d2_d4_d8_DSMB=1	*Convulsions in the previous 24 hrs*						
rct1_poor_clinical_oth_inf_DSMB=1	3. Another serious illness at day 2, 4 or 8, n (%)*<br>*signs not mutually exclusive*						
A3_meningitis_d2_d4_d8_DSMB=1	*Meningitis*						
A3_bone_d2_d4_d8_DSMB=1	*Bone or joint infection*						
A3_infection_other_d2_d4_d8_DSMB=1	*Other serious infection*						
rct1_poor_clinical_new_csi_DSMB=1	4. Any new signs of CSI at day 4 or 8, n (%)*<br>*signs not mutually exclusive*						
a3_chest_indraw_new_d4_d8_DSMB=1	*Severe chest indrawing*						
a3_high_body_temp_new_d4_d8_DSMB=1	*High body temperature (≥ 38C)*						
a3_fast_breath_new_d4_d8_DSMB=1	*Fast breathing ≥ 24 hours to < 7 days*						
a3_feed_diff_not_new_d4_d8_DSMB=1	*Stopped feeding well*						
a3_mov_only_stim_new_d4_d8_DSMB=1	*Movement only on stimulation*						
a3_low_body_temp_new_d4_d8_DSMB=1	*Low body temperature (<35.5C)*						

Variable name and value: Treatment variable rct1_trt_pp		Africa			Asia		
		**Total**	**Outpatient**	**Inpatient**	**Total**	**Outpatient**	**Inpatient**
rct1_poor_clinical_pers_csi_DSMB=1	5. Persistence of the low mort risk sign at day 8, n (%)* *signs not mutually exclusive*						
a3_chest_indrawing_pers_d8_DSMB=1	*Severe chest indrawing*						
a3_high_body_temp_pers_d8_DSMB=1	*High body temperature (≥ 38C)*						
a3_fast_breath_pers_d8_DSMB=1	*Fast breathing ≥ 24 hours to < 7 days*						

[1]Poor clinical outcome defined as: Death between randomisation and day 15 of initiation of therapy, or presence of any sign of critical illness (no movement at all, unable to feed at all, or convulsions) on day 2, 4 or day 8 of initiation of therapy, or any sign suggestive of another serious infection, e.g. meningitis, bone or joint infection, on day 2, 4 or day 8 of initiation of therapy, or presence of any new sign (same sign as screening at day 8 after disappearing on day 4, or a different sign from screening on day 4 or 8) of CSI on day 4 or day 8 of initiation of therapy, or persistence of the presenting sign on day 8 (same sign at screening, day 4 and day 8) of initiation of therapy. Subset to those randomised and at least 15 days from screening. LTF/withdrawn consent and have no information gathered about the infants' outcome on Days 2, 4 or 8.

*This is a participant-based analysis in which one infant is counted for one outcome in the hierarchical order of the report from the reasons listed from 1. Death through 5. Persistent sign. For example, if the infant has both a critical illness and another serious illness, then this infant is only counted once in critical illness. The percentage provided is for the total per-protocol population.

NOTES: Timing of death is available from the completed SAE forms. Signs under each outcome are not mutually exclusive, meaning that one infant can have more than one sign within each outcome. The proportions for each sign are among those with the given outcome.

Table 5. Summary of the infant's location during the trial and receipt of study medication by treatment group overall and stratified by region.

Variable name and value:<br>Treatment variable rct1_trt_itt		Overall	Africa	Asia
rct1_trt_itt=1	**Outpatient treatment group, n (%)**			
	Summary Treatment Information			
	Infant at home or came to hospital/clinic only for injection Days 1-7, n (%)			
a2_home_all_b=1	Yes			
a2_home_all_b=2	No			
	Initiated medication: 1+ dose injectable gentamicin on Day 1 and 1+ dose oral amoxicillin on Day 1			
a2_Outpatient_start_trt=1	Yes			
a2_Outpatient_start_trt=2	No			
a2_Outpatient_start_trt=3	Missing medication information			
	Received at least 80% of medication: 10+ doses of oral amoxicillin, 2+ doses of injectable gentamicin, and no other antibiotics for 7d			
a2_outpatient_80_trt=1	Yes			
a2_outpatient_80_trt=2	No			
a2_outpatient_80_trt=3	Missing medication information			
	Received all medication: 1+ dose injectable gentamicin on Days 1-2, 1+ dose oral amoxicillin on Day 1, and 2+ doses oral amoxicillin on Days 2-7, and no other antibiotics for 7d			
a2_Outpatient_full_trt=1	Yes			
a2_Outpatient_full_trt=2	No			
a2_Outpatient_full_trt=3	Missing medication information			
	Among those who did not receive a full course of treatment, n(%)			
a2_Outpatient_no_trt_cat=1	Did not receive any study treatment			
a2_Outpatient_no_trt_cat=2	Received partial study treatment			
a2_Outpatient_no_trt_cat=3	Received full study treatment but also received other antibiotics			

rct1_trt_itt=2	**Inpatient control group, n (%)**			
	Summary Treatment Information			
	Infant in hospital Days 1-7, n (%)			
a2_hosp_all_a=1	Yes			
a2_hosp_all_a=2	No			
	Initiated medication: 1+ dose injectable ampicillin on Day 1 and 1+ dose injectable gentamicin on Day 1			
a2_inpatient_start_trt=1	Yes			
a2_inpatient_start_trt=2	No			
a2_inpatient_start_trt=3	Missing medication information			
	Received at least 80% of medication: 5+ days of injectable ampicillin, 5+ doses of injectable gentamicin, and no other antibiotics for 7d			
a2_inpatient_80_trt=1	Yes			
a2_inpatient_80_trt=2	No			
a2_inpatient_80_trt=3	Missing medication information			
	Received all medication: 1+ dose injectable ampicillin on Day 1, 2+ doses injectable ampicillin on Days 2-6, 1+ doses injectable ampicillin on Day 7, 1+ dose injectable gentamicin for 7d, and no other antibiotics for 7d			
a2_inpatient_full_trt=1	Yes			
a2_inpatient_full_trt=2	No			
a2_inpatient_full_trt=3	Missing medication information			
	Among those who did not receive a full course of treatment, n(%)			
a2_inpatient_no_trt_cat=1	Did not receive any study treatment			
a2_inpatient_no_trt_cat=2	Received partial study treatment			
a2_inpatient_no_trt_cat=3	Received full study treatment but also received other antibiotics			

Table 6. Severe adverse events (excluding deaths) and treatment failure by treatment group overall and stratified by region.

Variable name and value: Treatment variable rct1_trt_itt		Overall			Africa			Asia		
		Total	Outpatient	Inpatient	Total	Outpatient	Inpatient	Total	Outpatient	Inpatient
RCT1_enrolled	**RCT 1 Enrolled, N**									
RCT1_any_SAE_exclude_deaths=1	SAE (excluding deaths) at any time from randomisation to day 15, n (%)									
	SAE (excluding deaths) related to treatment at any time from randomisation to day 15, n (%)									
rct1_SAE_relate_exclude_deaths=1	Not related									
rct1_SAE_relate_exclude_deaths=2	Unlikely related									
rct1_SAE_relate_exclude_deaths=3	Probably related									
rct1_SAE_relate_exclude_deaths=4	Highly probably related									
rct1_SAE_relate_exclude_deaths=5	It is not clear if it is related									
	**Treatment failure[1]**									
RCT1_outcome_trt_fail=1	Any failure noted, n (%)									
	Specific failure(s) – hierarchical, n (%)									
RCT1_outcome_trt_fail_cat=1	Death at any time from randomisation to day 15									
RCT1_outcome_trt_fail_cat=2	Anaphylactic reaction									

RCT1_outcome_trt_fail_cat=3	Severe diarrhoea									
RCT1_outcome_trt_fail_cat=4	Other, including disseminated or severe rash									

[1] Treatment failure includes any of the following: death, anaphylactic reaction, diarrhoea, and other likely allergic reactions, including disseminated or severe rash.

3.2. DRAFT FIGURES

Figure 1: Consort diagram of participant status

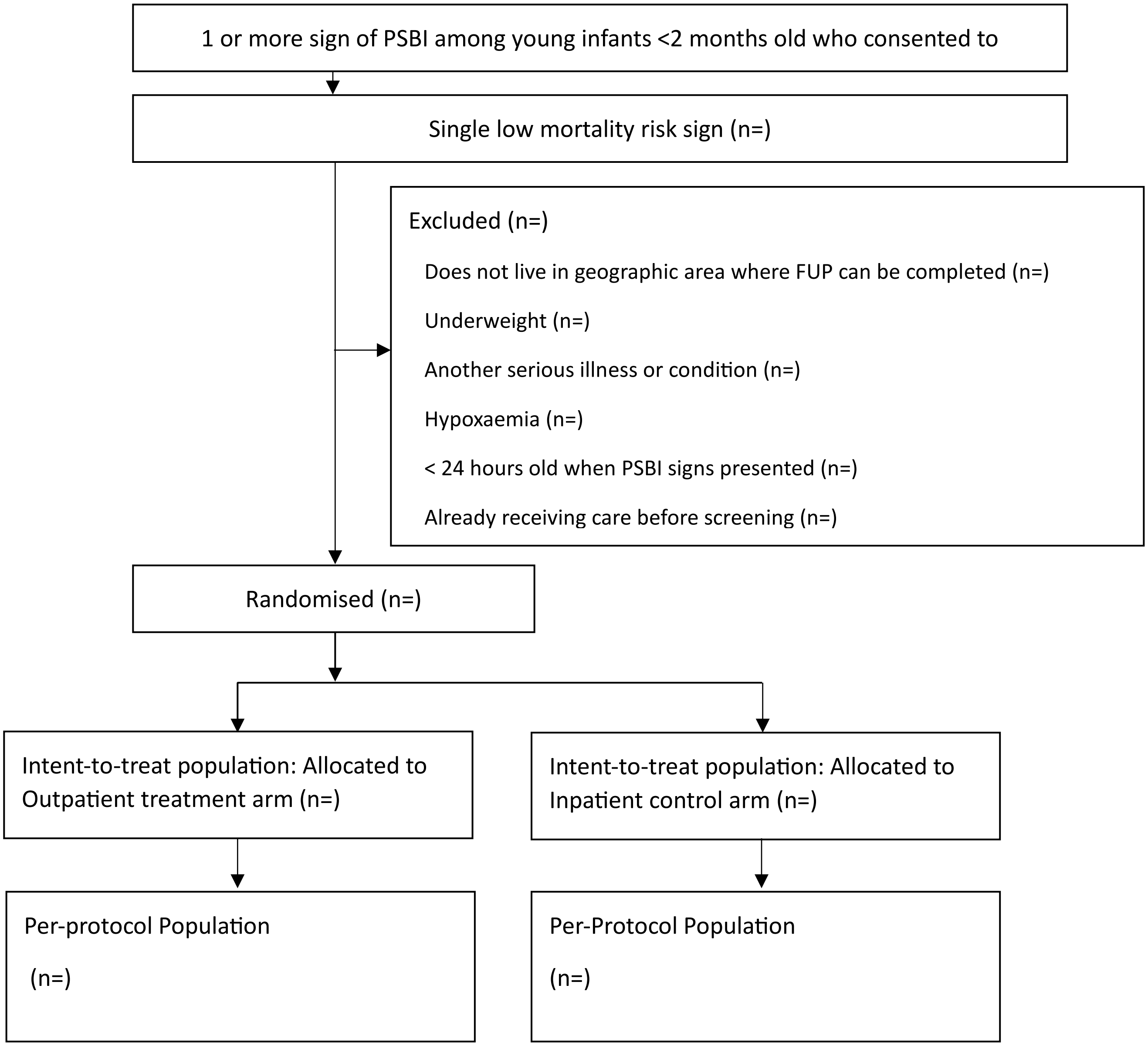

Figure 2. Superiority analysis of the primary and secondary outcomes and risk differences by treatment group among the overall intent-to-treat population.

Variable name and value:<br>Treatment variable rct1_trt_itt		Total	Outpatient	Inpatient	Superiority test p-value[2]	*[Forest plot of the risk difference with 95% CI. Ref line at zero.]*	Risk Difference (95% CI) [2]	Adjusted Risk Difference (95% CI) [2]
RCT1_enrolled	**Enrolled, N**				--		--	--
	Poor clinical outcome[1], n (%)							
rct1_poor_clinical_outcome_DSMB =1	Yes				--			
rct1_poor_clinical_outcome_DSMB =2	No				--		*Ref*	*Ref*
rct1_poor_clinical_outcome_DSMB =3	LFU / withdrew consent				--		*Ref*	*Ref*
	Individual components of poor clinical outcome				--		--	--
rct1_poor_clinical_death_DSMB=1	1. Death at any time from randomisation to day 15, n (%)*							
rct1_poor_clinical_crit_ill_DSMB=1	2. Critical illness day 2, 4 or 8, n (%)*<br>*signs not mutually exclusive*							
A3_infant_mov_none_d2_d4_d8_DSMB=1	*No movement at all*				--		--	--
a3_unable_feed_d2_d4_d8_DSMB=1	*Unable to feed at all*				--		--	--
A3_infant_fit_d2_d4_d8_DSMB=1	*Convulsions in the previous 24 hrs*				--		--	--

rct1_poor_clinical_oth_inf_DSMB=1	3. Another serious illness at day 2, 4 or 8, n (%)* *signs not mutually exclusive*						
A3_meningitis_d2_d4_d8_DSMB=1	*Meningitis*				--	--	--
A3_bone_d2_d4_d8_DSMB=1	*Bone or joint infection*				--	--	--
A3_infection_other_d2_d4_d8_DSMB=1	*Other serious infection*				--	--	--
rct1_poor_clinical_new_csi_DSMB=1	4. Any new signs of CSI at day 4 or 8, n (%)* *signs not mutually exclusive*						
a3_chest_indraw_new_d4_d8_DSMB=1	*Severe chest indrawing*				--	--	--
a3_high_body_temp_new_d4_d8_DSMB=1	*High body temperature (≥ 38C)*				--	--	--
a3_fast_breath_new_d4_d8_DSMB=1	*Fast breathing ≥ 24 hours to < 7 days*				--	--	--
a3_feed_diff_not_new_d4_d8_DSMB=1	*Stopped feeding well*				--	--	--
a3_mov_only_stim_new_d4_d8_DSMB=1	*Movement only on stimulation*				--	--	--
a3_low_body_temp_new_d4_d8_DSMB=1	*Low body temperature (<35.5C)*				--	--	--
rct1_poor_clinical_pers_csi_DSMB=1	5. Persistence of the low mort risk sign at day 8, n (%)* *signs not mutually exclusive*						

a3_chest_indrawing_pers_d8_DSMB=1	*Severe chest indrawing*				--		--	--
a3_high_body_temp_pers_d8_DSMB=1	*High body temperature (≥ 38C)*				--		--	--
a3_fast_breath_pers_d8_DSMB=1	*Fast breathing ≥ 24 hours to < 7 days*				--		--	--

[1]Poor clinical outcome defined as: Death between randomisation and day 15 of initiation of therapy, or presence of any sign of critical illness (no movement at all, unable to feed at all, or convulsions) on day 2, 4 or day 8 of initiation of therapy, or any sign suggestive of another serious infection, e.g. meningitis, bone or joint infection, on day 2, 4 or day 8 of initiation of therapy, or presence of any new sign (same sign as screening at day 8 after disappearing on day 4, or a different sign from screening on day 4 or 8) of CSI on day 4 or day 8 of initiation of therapy, or persistence of the presenting sign on day 8 (same sign at screening, day 4 and day 8) of initiation of therapy. Subset to those randomised and at least 15 days from screening. LTF/withdrawn consent and have no information gathered about the infants' outcome on Days 2, 4 or 8.

*This is a participant-based analysis in which one infant is counted for one outcome in the hierarchical order of the report from the reasons listed from 1. Death through 5. Persistent sign. For example, if the infant has both a critical illness and another serious illness, then this infant is only counted once in critical illness. The percentage provided is of the total intent-to-treat population.

NOTES: The timing of death is available from the completed SAE forms. Signs under each outcome are not mutually exclusive, meaning that one infant can have more than one sign within each outcome. The proportions for each sign are among those with the given outcome.

[2] The superiority of the study treatment is tested using a Farrington-Manning (1990) score test with a superiority margin of 30%, which compares the favourable outcome (i.e. those with either no poor clinical outcome or those lost to follow-up/withdrew consent) in the outpatient treatment arm to the inpatient control arm. The resulting test p-value is displayed. Risk differences and 95% confidence intervals for poor clinical outcomes are displayed numerically and in forest plots. The unadjusted and adjusted risk differences (and associated 95% CI) for poor clinical outcomes are obtained by fitting generalised linear models to experiencing a poor clinical outcome with the following fixed effects: treatment group and site (adjusted analysis only).

Figure 3. Non-inferiority analysis of the primary and secondary outcomes and risk differences by treatment group among the overall per-protocol population.

Variable name and value:<br>Treatment variable rct1_trt_pp		**Total**	**Outpatient**	**Inpatient**	**Non-inferiority test p-value[2]**	***[Forest plot of the risk difference with 95% CI. Ref line at zero.]***	**Risk Difference (95% CI) [2]**	**Adjusted Risk Difference (95% CI) [2]**
RCT1_enrolled	**Enrolled, N**				--		--	--
	Poor clinical outcome[1], n (%)						--	--
rct1_poor_clinical_outcome_DSMB=1	Yes				--			
rct1_poor_clinical_outcome_DSMB=2	No				--		*Ref*	*Ref*
	Individual components of poor clinical outcome				--		--	--
rct1_poor_clinical_death_DSMB=1	1. Death at any time from randomisation to day 15, n (%)*							
rct1_poor_clinical_crit_ill_DSMB=1	2. Critical illness day 2, 4 or 8, n (%)*<br>*signs not mutually exclusive*							
A3_infant_mov_none_d2_d4_d8_DSMB=1	*No movement at all*				--		--	--
a3_unable_feed_d2_d4_d8_DSMB=1	*Unable to feed at all*				--		--	--
A3_infant_fit_d2_d4_d8_DSMB=1	*Convulsions in the previous 24 hrs*				--		--	--

rct1_poor_clinical_oth_inf_DSMB=1	3. Another serious illness at day 2, 4 or 8, n (%)*<br>*signs not mutually exclusive*						
A3_meningitis_d2_d4_d8_DSMB=1	*Meningitis*				--	--	--
A3_bone_d2_d4_d8_DSMB=1	*Bone or joint infection*				--	--	--
A3_infection_other_d2_d4_d8_DSMB=1	*Other serious infection*				--	--	--
rct1_poor_clinical_new_csi_DSMB=1	4. Any new signs of CSI at day 4 or 8, n (%)*<br>*signs not mutually exclusive*						
a3_chest_indraw_new_d4_d8_DSMB=1	*Severe chest indrawing*				--	--	--
a3_high_body_temp_new_d4_d8_DSMB=1	*High body temperature (≥ 38C)*				--	--	--
a3_fast_breath_new_d4_d8_DSMB=1	*Fast breathing ≥ 24 hours to < 7 days*				--	--	--
a3_feed_diff_not_new_d4_d8_DSMB=1	*Stopped feeding well*				--	--	--
a3_mov_only_stim_new_d4_d8_DSMB=1	*Movement only on stimulation*				--	--	--
a3_low_body_temp_new_d4_d8_DSMB=1	*Low body temperature (<35.5C)*				--	--	--

rct1_poor_clinical_pers_csi_DSMB=1	5. Persistence of the low mort risk sign at day 8, n (%)* *signs not mutually exclusive*							
a3_chest_indrawing_pers_d8_DSMB=1	*Severe chest indrawing*				--		--	--
a3_high_body_temp_pers_d8_DSMB=1	*High body temperature (≥ 38C)*				--		--	--
a3_fast_breath_pers_d8_DSMB=1	*Fast breathing ≥ 24 hours to < 7 days*				--		--	--

[1]Poor clinical outcome defined as: Death between randomisation and day 15 of initiation of therapy, or presence of any sign of critical illness (no movement at all, unable to feed at all, or convulsions) on day 2, 4 or day 8 of initiation of therapy, or any sign suggestive of another serious infection, e.g. meningitis, bone or joint infection, on day 2, 4 or day 8 of initiation of therapy, or presence of any new sign (same sign as screening at day 8 after disappearing on day 4, or a different sign from screening on day 4 or 8) of CSI on day 4 or day 8 of initiation of therapy, or persistence of the presenting sign on day 8 (same sign at screening, day 4 and day 8) of initiation of therapy. Subset to those randomised and at least 15 days from screening. LTF/withdrawn consent and have no information gathered about the infants' outcome on Days 2, 4 or 8.

*This is a participant-based analysis in which one infant is counted for one outcome in the hierarchical order of the report from the reasons listed from 1. Death through 5. Persistent sign. For example, if the infant has both a critical illness and another serious illness, then this infant is only counted once in critical illness. The percentage provided is of the total per-protocol population.

NOTES: The timing of death is available from completed SAE forms. Signs under each outcome are not mutually exclusive, meaning that one infant can have more than one sign within each outcome. The proportions for each sign are among those with the given outcome.

[2] The non-inferiority of the study treatment is tested using a Farrington-Manning (1990) score test with a non-inferiority margin of 1.8% which compares the favourable outcome (i.e. no poor clinical outcome) in the outpatient treatment to the inpatient control group. The resulting test p-value is displayed. Risk differences and 95% confidence intervals for poor clinical outcomes are displayed numerically and in forest plots. The unadjusted and adjusted risk differences (and associated 95% CI) for poor clinical outcomes are obtained by fitting generalised linear models to experiencing a poor clinical outcome with the following fixed effects: treatment group and site (adjusted analysis only).

Repeat tables by site (Supplement Tables 1-6)

SECTION 4. TIMELINE

- Complete revision of table shells and analysis plan – May 6, 2024
- Completion of the initial analyses. – Draft May 28, 2024 (Draft using the May data lock. Rerun once all enrolments and follow-ups are completed, as well as the edits and sites have confirmed numbers.)
- Draft papers to co-authors – June/July depending on final data collection
- Revise papers (as needed) based on co-author feedback – July/August
- Submit for final WHO clearance and then to journal - August

SECTION 5. REFERENCES

1 United Nations Inter-agency Group for Child Mortality Estimation (UN IGME). Levels and Trends in Child Mortality: Report 2022. New York: United Nations Children's Fund; 2022. Available: https://data.unicef.org/resources/levels-and-trends-in-child-mortality/. Accessed February 8 2023.
2 Alliance for Maternal and Newborn Health Improvement (AMANHI) mortality study group. Population-based rates, timing, and causes of maternal deaths, stillbirths, and neonatal deaths in south Asia and sub-Saharan Africa: a multi-country prospective cohort study. Lancet Glob Health. 2018;6:e1297-308. Medline:30361107 doi:10.1016/S2214-109X(18)30385-1
3 World Health Organization. Integrated Management of Childhood Illness: management of the sick young infant aged up to 2 months. IMCI chart booklet. Geneva, Switzerland: WHO; 2019. Available: https://www.who.int/publications/i/item/9789241516365. Accessed February 8 2023.
4 World Health Organization. Pocket book of hospital care for children: guidelines for the management of common childhood illnesses. 2nd ed. Switzerland: World Health Organization; 2013. Available at: https://apps.who.int/iris/bitstream/handle/10665/81170/9789241548373_eng.pdf?sequence=1. Accessed February 8 2023.
5 World Health Organization. Guideline: Managing possible serious bacterial infection in young infants when referral is not feasible. Switzerland: WHO; 2015. Available: https://www.who.int/publications/i/item/9789241509268. Accessed February 8 2023.
6 Tshefu A, Lokangaka A, Ngaima S, Engmann C, Esamai F, Gisore P, et al. Simplified antibiotic regimens compared with injectable procaine benzylpenicillin plus gentamicin for treatment of neonates and young infants with clinical signs of possible serious bacterial infection when referral is not possible: a randomised, open-label, equivalence trial. Lancet. 2015;385:1767-76. Medline:25842221 doi:10.1016/S0140-6736(14)62284-4
7 Tshefu A, Lokangaka A, Ngaima S, Engmann C, Esamai F, Gisore P, et al. Oral amoxicillin compared with injectable procaine benzylpenicillin plus gentamicin for treatment of neonates and young infants with fast breathing when referral is not possible: a randomised, open-label, equivalence trial. Lancet. 2015;385:1758-66. Medline:25842223 doi:10.1016/ S0140-6736(14)62285-6
8 Baqui AH, Saha SK, Ahmed AS, Shahidullah M, Quasem I, Roth DE, et al. Safety and efficacy of alternative antibiotic regimens compared with 7 day injectable procaine benzylpenicillin and gentamicin for outpatient treatment of neonates and young infants with clinical signs of severe infection when referral is not possible: a randomised, open-label, equivalence trial. Lancet Glob Health. 2015;3:e279-87. Medline:25841891 doi:10.1016/S2214-109X(14)70347-X
9 Mir F, Nisar I, Tikmani SS, Baloch B, Shakoor S, Jehan F, et al. Simplified antibiotic regimens for treatment of clinical severe infection in the outpatient setting when referral is not possible for young infants in Pakistan (Simplified Antibiotic Therapy Trial [SATT]): a randomised, open-label, equivalence trial. Lancet Glob Health. 2017;5:e177-85. Medline:27988146 doi:10.1016/S2214-109X(16)30335-7
10 Wammanda RD, Adamu SA, Joshua HD, Nisar YB, Qazi SA, Aboubaker S, et al. Implementation of the WHO guideline on treatment of young infants with signs of possible serious bacterial infection when hospital referral is not feasible in rural Zaria, Nigeria: Challenges and solutions. PLoS One. 2020;15:e0228718. Medline:32155155 doi:10.1371/journal.pone.0228718
11 Guenther T, Mopiwa G, Nsona H, Qazi S, Makuluni R, Fundani CB, et al. Feasibility of implementing the World Health Organization case management guideline for possible serious bacterial infection among young infants in Ntcheu district, Malawi. PloS one. 2020;15:e0229248. Medline:32287262 doi:10.1371/journal.pone.0229248
12 Rahman AE, Herrera S, Rubayet S, Banik G, Hasan R, Ahsan Z, et al. Managing possible serious bacterial infection of young infants where referral is not possible: Lessons from the early implementation experience in Kushtia District learning laboratory, Bangladesh. PLoS One. 2020;15:e0232675. Medline:32392209 doi:10.1371/journal.pone.0232675
13 Awasthi S, Kesarwani N, Verma RK, Agarwal GG, Tewari LS, Mishra RK, et al. Identification and management of young infants with possible serious bacterial infection where referral was not feasible in rural Lucknow district of Uttar Pradesh, India: An implementation research. PLoS One. 2020;15:e0234212. Medline:32497092 doi:10.1371/journal.pone.0234212
14 Roy S, Patil R, Apte A, Thibe K, Dhongade A, Pawar B, et al. Feasibility of implementation of simplified management of young infants with possible serious bacterial infection when referral is not feasible in tribal areas of Pune district, Maharashtra, India. PLoS One. 2020;15:e0236355. Medline:32833993 doi:10.1371/journal.pone.0236355
15 Goyal N, Rongsen-Chandola T, Sood M, Sinha B, Kumar A, Qazi SA, et al. Management of possible serious bacterial infection in young infants closer to home when referral is not feasible: Lessons from implementation research in Himachal Pradesh, India. PLoS One. 2020;15:e0243724. Medline:33351810 doi:10.1371/journal.pone.0243724
16 Leul A, Hailu T, Abraham L, Bayray A, Terefe W, Godefay H, et al. Innovative approach for potential scale-up to jump-start simplified management of sick young infants with possible serious bacterial infection when a referral is not feasible: Findings from implementation research. PLoS One. 2021;16:e0244192. Medline:33544712 doi:10.1371/journal.pone.0244192
17 Mukhopadhyay R, Arora NK, Sharma PK, Dalpath S, Limbu P, Kataria G, et al. Lessons from implementation research on community management of Possible Serious Bacterial Infection (PSBI) in young infants (0-59 days), when the referral is

not feasible in Palwal district of Haryana, India. PLoS One. 2021;16:e0252700. Medline:34234352 doi:10.1371/journal.pone.0252700
18 Ayede AI, Ashubu OO, Fowobaje KR, Aboubaker S, Nisar YB, Qazi SA, et al. Management of possible serious bacterial infection in young infants where referral is not possible in the context of existing health system structure in Ibadan, South-west Nigeria. PLoS One. 2021;16:e0248720. Medline:33784321 doi:10.1371/journal.pone.0248720
19 Applegate JA, Ahmed S, Khan MA, Alam S, Kabir N, Islam M, et al. Early implementation of guidelines for managing young infants with possible serious bacterial infection in Bangladesh. BMJ Glob Health. 2019;4:e001643. Medline:31803507 doi:10.1136/bmjgh-2019-001643

20 Berhane M, Girma T, Tesfaye W, Jibat N, Abera M, Abrahim S, et al. Implementation research on management of sick young infants with possible serious bacterial infection when referral is not possible in Jimma Zone, Ethiopia: challenges and solutions. PLoS One. 2021;16:e0255210. Medline:34370744 doi:10.1371/journal.pone.0255210
21 Ariff S, Soofi SB, Suhag Z, Chanar S, Bhura M, Dahar Z, et al. Implementation research to increase treatment coverage of possible serious bacterial infections in young infants when a referral is not feasible: lessons learnt. J Public Health (Oxf.). 2023;45:176-188. Medline:35138390 doi:10.1093/pubmed/fdab409
22 Lokangaka A, Ishoso D, Tshefu T, Kalonji M, Takoy P, Kokolomami J, et al. Simplified antibiotic regimens for young infants with possible serious bacterial infection when the referral is not feasible in the Democratic Republic of the Congo. PLoS One. 2022;17: e0268277. Medline:35771738 doi:10.1371/journal.pone.0268277
23 Nisar YB, Aboubaker S, Arifeen SE, Ariff S, Arora N, Awasthi S, et al. A multi-country implementation research initiative to jump-start scale-up of outpatient management of possible serious bacterial infections (PSBI) when a referral is not feasible: summary findings and implications for programs. In press. 2022.
24 Sands K, Carvalho MJ, Portal E, Thomson K, Dyer C, Akpulu C, et al. Characterisation of antimicrobial-resistant Gram-negative bacteria that cause neonatal sepsis in seven low- and middle-income countries. Nat Microbiol. 2021;6:512-23. Medline:33782558 doi:10.1038/s41564-021-00870-7
25 Dramowski A, Madide A, Bekker A. Neonatal nosocomial bloodstream infections at a referral hospital in a middle-income country: burden, pathogens, antimicrobial resistance and mortality. Paediatr Int Child Health. 2015;35:265-72. Medline:25940506 doi:10.1179/2046905515Y.0000000029
26 Maoulainine FM, Elidrissi NS, Chkil G, Abba F, Soraa N, Chabaa L, et al. [Epidemiology of nosocomial bacterial infection in a neonatal intensive care unit in Morocco]. Arch Pediatr. 2014;21:938-43. Medline:24993147 doi:10.1016/j.arcped.2014.04.033
27 Shahunja KM, Ahmed T, Faruque AS, Shahid AS, Das SK, Shahrin L, et al. Experience With Nosocomial Infection in Children Under 5 Treated in an Urban Diarrheal Treatment Center in Bangladesh. Glob Pediatr Health. 2016;3:X16634267. Medline:27336005 doi:10.1177/2333794X16634267
28 Nisar YB, Tshefu A, Longombe AL, Esamai F, Marete I, Ayede AI, et al. Clinical signs of possible serious infection and associated mortality among young infants presenting at first-level health facilities. PLoS One. 2021;16:e0253110. Medline:34191832 doi:10.1371/journal.pone.0253110
29 International Standard Randomised Controlled Trial Number (ISRCTN) Registry. How long should young infants less than 2 months of age with moderate-mortality-risk signs of possible serious bacterial infection be hospitalised for? 2021.
30 Kozuki N, Guenther T, Vaz L, Moran A, Soofi SB, Kayemba CN, et al. A systematic review of community-to-facility neonatal referral completion rates in Africa and Asia. BMC Public Health. 2015;15:989. Medline:26419934 doi:10.1186/s12889-015-2330-0
31 Applegate JA, Ahmed S, Harrison M, Callaghan-Koru J, Mousumi M, Begum N, et al. Caregiver acceptability of the guidelines for managing young infants with possible serious bacterial infections (PSBI) in primary care facilities in rural Bangladesh. PLoS One. 2020;15:e0231490. Medline:32287286 doi:10.1371/journal.pone.0231490
32 Teklu AM, Litch JA, Tesfahun A, Wolka E, Tuamay BD, Gidey H, et al. Referral systems for preterm, low birth weight, and sick newborns in Ethiopia: a qualitative assessment. BMC Pediatr. 2020;20:409. Medline:32861246 doi:10.1186/s12887-020-02311-6
33 Owais A, Sultana S, Stein AD, Bashir NH, Awaldad R, Zaidi AK. Why do families of sick newborns accept hospital care? A community-based cohort study in Karachi, Pakistan. J Perinatol. 2011;31:586-92. Medline:21273989 doi:10.1038/ jp.2010.191

TABLE SM3: POOR CLINICAL OUTCOME USING INTENT-TO-TREAT POPULATION, ADJUSTED BY THE STUDY SITE (N=7001)

Outcome	Outpatient (n=3501)	Inpatient (n=3500)	Adjusted risk difference (95% CI)
Poor clinical outcome, n (%)			
Yes	269 (7·7)	272 (7·8)	0·0004 (-0·0101, 0·0110)
No	3206 (91·6)	3159 (90·3)	*Ref*
LFU/withdrawn consent	26 (0·7)	69 (2·0)	*Ref*

TABLE SM4: POOR CLINICAL OUTCOME USING PER-PROTOCOL POPULATION, ADJUSTED BY THE STUDY SITE (N=6871)

Outcome	Outpatient (n=3455)	Inpatient (n=3416)	Adjusted risk difference (95% CI)
Poor clinical outcome, n (%)			
Yes	266 (7·7)	269 (7·9)	-0·0004 (-0·0110, 0·0102)
No	3189 (92·3)	3147 (92·1)	*Ref*

TABLE SM5: REASONS FOR LOSS TO FOLLOW-UP/WITHDRAWAL FROM THE STUDY BY TREATMENT ARM (N=95)

Reasons for loss to follow-up/withdrawal from the study	Outpatient arm (n=26) N (%)	Inpatient arm (n=69) N (%)
Parents refused admission/stay in the hospital/treatment without stating any reason	6 (23.1%)	45 (65.2%)
Parents took their young infant to another hospital/doctor as the infant had no improvement	7 (26.9%)	12 (17.4%)
The study team was unable to contact the parents	8 (30.8%)	4 (5.8%)
Parents refused admission/stay in the hospital due to the mother's health, or no one was available to take care of the other children at home.	NA	3 (4.3%)
Parents left the treatment/hospitalisation as the young infant had improved	2 (7.7%)	3 (4.3%)
Parents moved to another city/outside the study catchment area	3 (11.5%)	2 (2.9%)

TABLE SM6: COMPARISON OF BASELINE CHARACTERISTICS OF YOUNG INFANTS WHO WERE LOST TO FOLLOW-UP/WITHDRAWAL AND THOSE WHO WERE NOT LOST TO FOLLOW-UP/WITHDRAWAL FROM THE STUDY BY TREATMENT ARM

	Outpatient treatment arm (n=3501)			Inpatient treatment arm (n=3500)		
	Not withdrawal/LTFU (n=3475) N (%)	Withdrawal/ LTFU (n=26) N (%)	p-value	Not withdrawal/LTFU (n=3431) N (%)	Withdrawal/LTFU (n=69) N (%)	p-value
Sex			0.147			0.626
Male	2043 (58.8%)	19 (73.1%)		2139 (62.3%)	45 (65.2%)	
Female	1432 (41.2%)	7 (26.9%)		1292 (37.7%)	24 (34.8%)	
Age (days)			0.006			0.095
≥24 hours to < 7 days old	968 (27.9%)	1 (3.8%)		1020 (29.7%)	12 (17.4%)	
7-28 days old	1131 (32.5%)	9 (34.6%)		1117 (32.6%)	28 (40.6%)	
29-59 days old	1376 (39.6%)	16 (61.5%)		1294 (37.7%)	29 (42.0%)	
Weight-for-age (z-score) (WAZ)			0.003			0.807
< -2	627 (18.0%)	10 (38.5%)		662 (19.3%)	12 (17.4%)	
-2 to <-1	1113 (32.0%)	10 (38.5%)		1099 (32.0%)	22 (31.9%)	
-1 to 0	1194 (34.4%)	5 (19.2%)		1086 (31.6%)	28 (40.6%)	
> 0	541 (15.6%)	1 (3.8%)		584 (17.0%)	7 (10.1%)	
Single Presenting Clinical Sign			0.027			0.007
Severe chest indrawing	1354 (39.0%)	15 (57.7%)		1394 (40.6%)	42 (60.9%)	
High body temperature (≥ 38C)	1808 (52.0%)	11 (42.3%)		1704 (49.7%)	21 (30.4%)	
Fast breathing ≥24 hours to <7 days old	313 (9.1%)	0 (0.0%)		333 (9.7%)	6 (8.7%)	

TABLE SM7. NUMBER (%) YOUNG INFANTS FOLLOWED ON DAY 2, 4, 8 AND 15 BY TREATMENT ARM

Visit day	Outpatient (n=3501)<br>N (%)	Inpatient (n=3500)<br>N (%)
Day 2	3476 (99·3%)	3488 (99·7%)
Day 4	3446 (98·4%)	3480 (99·4%)
Day 8	3419 (97·7%)	3472 (99·2%)
Day 15	3413 (97·5%)	3467 (99·1%)

**Full list of PSBI Study Group (alphabetically country-based):**
Bangladesh site: Abdullah H Baqui (Johns Hopkins Bloomberg School of Public Health, Baltimore, Maryland, USA); Mohammod Shahidullah, (Bangabandhu Sheikh Mujib Medical University, Dhaka, Bangladesh); Salahuddin Ahmed (Projahnmo Research Foundation, Dhaka, Bangladesh); Arunangshu Dutta Roy (Projahnmo Research Foundation, Dhaka, Bangladesh); Rasheda Khanam (Johns Hopkins Bloomberg School of Public Health, Baltimore, Maryland, USA); Iffat Ara Jaben (Projahnmo Research Foundation, Dhaka, Bangladesh); Nabidul Haque Chowdhury, (Projahnmo Research Foundation, Dhaka, Bangladesh); Sabina Ashrafee Lipi (National Newborn Health Services, Directorate General of Health Services, Dhaka, Bangladesh); Md Jahurul Islam (National Newborn Health Services, Directorate General of Health Services, Dhaka, Bangladesh); Manajjir Ali (Projahnmo Research Foundation, Dhaka, Bangladesh).<br><br>Ethiopia site: Amha Mekasha, Abiy Seifu Estifanos, Lulu Muhe, Damen Hailemariam, Dorka Woldesenbet Keraga, Tabot Keskis Azeze (Addis Ababa University, Addis Ababa, Ethiopia); Bogale Worku (Ethiopian Pediatric Society, Addis Ababa, Ethiopia); Solome Jebessa (St. Hospital millennium medical college, Addis Ababa, Ethiopia).<br><br>India site 1: Temsunaro Rongsen-Chandola, Nidhi Goyal, Amit Kumar, Nita Bhandari, Uma Chandra Mouli Natchu, Manisha Gupta, Aritra Guha, (Society for Applied Studies, New Delhi, India); Shayam Kaushik, Surjeet Kumar (Dr YS Parmar Government Medical College, Nahan, Himachal Pradesh, India); Amitabh Jain (Civil Hospital Paonta Sahib, Sirmaur, Himachal Pradesh, India); Mangla Sood, Rakesh Sharma (Indira Gandhi Medical College, Shimla, Himachal Pradesh, India); Jagjit Singh Dalal, Kundan Mittal (Pt. BD Sharma Post Graduate Institute of Medical Sciences, Medical Road, Rohtak, Haryana, India); GP Kaushal, Vineeta Wadhwa (Dr. BS Ambedkar Hospital, Rohini, New Delhi, India); Anju Seth, Varinder Singh, Harish Pemde, Praveen Kumar, Viswas Chhapola (Lady Hardinge Medical College and Associated Hospitals, New Delhi, India).<br><br>India site 2: Yashwant Kumar Rao, Arun Kumar Arya (GSVM Medical College, Kanpur, India); Krishna Kumar Dokania (Shyam Children & Maternity Centre, Kanpur, India); Ved Prakash (Directorate of Medical & Health Services, Government of Uttar Pradesh, India); Shakal Narayan Singh (King George's Medical University, Lucknow, India); Neeraj Kumar (SN Medical College, Agra, India); Shiv Kumar (AHM & Dufferin District Women's Hospital, Kanpur, India); Vinay Pratap Singh, Pramod Kumar Singh, Vivek Kumar Singh, Rashmi Kumar, Aarti Kumar, Vishwajeet Kumar (Community Empowerment Lab, Lucknow, India).<br><br>Nigeria site: Robinson Daniel Wammanda, Laila Hassan (Department of Paediatrics, Ahmadu Bello University Teaching Hospital, Zaria, Nigeria); Saraja Ahmodu Opaluwa (Department of Medical Microbiology, Ahmadu Bello University Teaching Hospital, Zaria, Nigeria); Ishaku Hassan (Department of Paediatrics, Ahmadu Bello University Teaching Hospital, Zaria, Nigeria); Aminu Shadrach Adamu (Department of Community Medicine, Ahmadu Bello University Teaching Hospital, Zaria, Nigeria); Bawa Ega (Department of Medical Microbiology, Ahmadu Bello University Teaching Hospital, Zaria, Nigeria); Daniel Efemena Atinaya (AFRINEST Research Unit, Ahmadu Bello University Teaching Hospital, Zaria).<br><br>Pakistan site: Fyezah Jehan (Aga Khan University, Karachi, Pakistan), Imran Nisar (Aga Khan University, Karachi, Pakistan), Benazir Baloch (Aga Khan University, Karachi, Pakistan), Dania Omer Ansari (Aga Khan University, Karachi, Pakistan), Kiran Lalani (Aga Khan University, Karachi, Pakistan), Najeeb

Rehman (Aga Khan University, Karachi, Pakistan), Azhar Raza (Aga Khan University, Karachi, Pakistan), Tooba Ahmed Alvi (Aga Khan University, Karachi, Pakistan), Salman Osmani (Aga Khan University, Karachi, Pakistan), Aneeta Hotwani (Aga Khan University, Karachi, Pakistan), Erum Salman (Aga Khan University, Karachi, Pakistan), Suneeta Namdave (Aga Khan University, Karachi, Pakistan), Muhammad Hanif (National Institute of Child Health, Karachi, Pakistan), Nasir Saleem Saddal (National Institute of Child Health, Karachi, Pakistan), Jamal Raza (Sindh Institute of Child Health and Neonatology, Karachi, Pakistan), Syed Rehan Ali (Sindh Institute of Child Health and Neonatology, Karachi, Pakistan), Shahid Raza (Sindh Institute of Child Health and Neonatology, Karachi, Pakistan), Muhammad Tofique (Sindh Government children Hospital), Mashal Khan (National Institute of Child Health, Karachi, Pakistan), Wajid Hussain (National Institute of Child Health, Karachi, Pakistan), Muhammad Hayat Bozdar (National Institute of Child Health, Karachi, Pakistan), Mahmood Shaikh (National Institute of Child Health, Karachi, Pakistan), Musarat Ayaz (National Institute of Child Health, Karachi, Pakistan), Sumaira Wajid (National Institute of Child Health, Karachi, Pakistan), Serajunissa Syed, (National Institute of Child Health, Karachi, Pakistan), Muhammad Naveed (National Institute of Child Health, Karachi, Pakistan), Hassan Abdul Jabbar (Sindh Institute of Child Health and Neonatology, Karachi, Pakistan), Azeem Khan (Sindh Institute of Child Health and Neonatology, Karachi, Pakistan).

Tanzania site: Karim Manji (Department of Pediatrics, Muhimbili University of Health and Allied Sciences, Dar-es-Salaam, Tanzania); Christopher R. Sudfeld (Harvard School of Public Health, Boston, Massachusetts, USA); Rodrick Kisenge, Nahya Salim, Sarah Somji, Mohamed Kheri Bakari, Fatimah Dhallah , Fred Maleko , Kristina Lugangira, Veneranda M Ndensangia (Department of Pediatrics, Muhimbili University of Health and Allied Sciences, Dar-es-Salaam, Tanzania); Christopher P. Duggan (Division of Gastroenterology, Hepatology and Nutrition, Boston Children's Hospital, Boston, Massachusetts, USA).

Data Management centre: Vanessa Thorsten; Elizabeth McClure; Norman Goco; David Plotner; Barbara Do; Norma Pugh, Melissa Page (RTI International, USA).

Trial Coordination Team: Sachiyo Yoshida (Department of Sexual, Reproductive, Maternal, Child and Adolescent Health and Ageing: Advancing Life Course Health and Reproduction (LHR), World Health Organization, Geneva, Switzerland); Shamim A Qazi (Independent Newborn and Child Consultant, Switzerland); Rajiv Bahl (ICMR, India); Yasir Bin Nisar (Department of Sexual, Reproductive, Maternal, Child and Adolescent Health and Ageing: Advancing Life Course Health and Reproduction (LHR), World Health Organization, Geneva, Switzerland).
