# Supplementary material for: Inpatient versus outpatient management of young infants with a single low-mortality-risk sign of possible serious bacterial infection in sub-Saharan Africa and south Asia: an open-label, multicentre, two-arm, randomised controlled trial
Source: Lancet Glob Health. 2025 Oct 15;13(11):e1892–902. doi: 10.1016/S2214-109X(25)00243-8 (PMC12535820; doi:10.1016/S2214-109X(25)00243-8)
Supplement: Equitable Partnership Declaration [file mmc2.pdf]

THE LANCET
Global Health

Supplementary appendix 2

This Equitable Partnership Declaration (EPD) was submitted by the authors, and we reproduce it as supplied. It has not been peer reviewed. *The Lancet*'s editorial processes have not been applied to the EPD.

Supplement to: PSBI Study Group. Inpatient versus outpatient management of young infants with a single low-mortality-risk sign of possible serious bacterial infection in sub-Saharan Africa and south Asia: an open-label, multicentre, two-arm, randomised controlled trial. *Lancet Glob Health* 2025; **13:** e1892–902.

Equitable Partnership Declaration

If any questions do not apply to your study, please indicate "N/A" for "not applicable.
For more information on how to complete this form see XXXXXX [IfA]

Researcher considerations

1. Please detail the involvement that researchers who are based in the country or countries of study had during a) study design; b) clinical study processes, such as processing blood samples, prescribing medication, or patient recruitment; c) data interpretation; and d) manuscript preparation, commenting on all aspects. If they were not involved in any of these aspects, please explain why.

   This should include a thorough description of their leadership roles in the study. Are local researchers named in the author list or the acknowledgements, or are they not mentioned at all (and, if not, why)? Please also describe the involvement of early career researchers based in the location of the study. Some of this information might be repeated from the Contributors section in the manuscript. Note: we adhere to ICMJE authorship criteria for naming authors on a paper.

**a) Study design:**<br>Local PIs of the trial from Bangladesh, Ethiopia, India (two sites), Nigeria, Pakistan, and Tanzania, who are themselves based in the country of the study, were involved in the design of the study. WHO facilitated a protocol development workshop in Geneva, and study site PIs participated in developing the trial protocol.
**b) Clinical study processes:**<br>Local PIs, research team and participating hospital staff at each trial site were involved in the clinical study processes, such as recruitment of the patients, treatment and outcome assessment, supportive supervision and monitoring.<br>Local PIs held leadership roles as site overseers. Early career researchers likely participated in operational roles within study teams.
**c) Data interpretation:**<br>Local PIs and teams (research and hospital staff) at each trial site worked with the primary analysis team on data analysis and interpretation. WHO held a five-day workshop in Tanzania, where study site PIs and core members jointly interpreted data and drafted the manuscript.
**d) Manuscript preparation:**<br>Local PIs and their research and clinical teams at each site contributed to preparing the manuscript. WHO organised a 5-day data analysis and writing workshop in Tanzania, attended by study site PIs and their core teams to develop the manuscript.

2. How was funding used to remunerate and enhance the skills of researchers in the countries of study? And how was funding used to improve research infrastructure at the study sites?

   Potentially effective investments into long-term skills and opportunities within local institutions could include training or mentorship in analytical techniques and manuscript writing,

opportunities to lead all or specific aspects of the study, financial remuneration rather than requiring volunteers, and other professional development and educational opportunities.

Improvements to research infrastructure could include funding extended trial designs (eg, platform trials), establishment of long-term contracts for research staff, building research facilities, and setting up local control of funding allocation.

**Skills:**<br>Funding supported training in PSBI assessment, IMCI protocols, and SOPs, with quarterly standardisation exercises, refresher training, and WHO oversight via monthly reviews and site visits.
**Research infrastructure:**<br>Funding supported the development of research infrastructure at certain sites. For instance, new PSBI management wards were built in Bangladesh and India's UP site to increase hospital capacity. Research offices were also established at all participating hospitals, strengthening infrastructure at public sector facilities.

3. How did you safeguard the researchers who implemented the study?

Please describe how you guaranteed safe working conditions for study staff, including provision of appropriate personal protective equipment, protection from violence, and prevention of overworking.

Researcher safety was ensured throughout the implementation of the trial. Standard hospital safety protocols, including PPE, especially during the COVID-19 pandemic, were provided to the research staff. Local PIs monitored researchers' well-being by following all institutional guidelines and ethical codes.

Benefits to the communities and regions of study

4. How does the study address the research and policy priorities of its location?

How were the local priorities determined and then used to inform the research question? Who decided which priorities to take forward? Which elements of the study address those priorities?

Before the study was designed, the WHO organised a three-day meeting that brought together over 30 experts from low- and middle-income countries. The purpose of this gathering was to discuss key issues related to the management of newborn and young infant infections (particularly PSBI). During the meeting, several important topics were addressed, and participants used a voting process to identify the most pressing research priorities. As a result, two critical research questions emerged and were further developed into concept notes for clinical trials—one of which became the basis for the current study. This collaborative and systematic approach ensured that the study addressed locally relevant

policy and research priorities, as determined and agreed upon by a diverse group of regional experts.

5. How will research products be shared in the community of study?

For instance, will you be providing written or oral layperson summaries for non-academic information sharing? Will study data be made available to institutions in the region(s) of study? The Lancet Global Health *encourages authors to translate the summary (abstract) into relevant languages after paper editing; do you intend to translate your summary?*

To ensure effective dissemination of research findings within the study community, PIs, in collaboration with the WHO, will organise dedicated dissemination meetings at each study site. These gatherings will engage not only healthcare professionals and local stakeholders but will also include local journalists to promote broader public awareness. Efforts will be made to present the study's results in clear, accessible formats, such as written and oral summaries designed for lay audiences. Additionally, findings will be shared through local newspapers and community channels to reach non-academic audiences. Where feasible, study summaries will be translated into relevant local languages to maximise accessibility. Study data will also be made available to regional institutions to support ongoing policy-making and research initiatives, aligning with The Lancet Global Health's encouragement for authors to provide translated abstracts following publication.

6. How were individuals, communities, and environments protected from harm?

a) *How did you ensure that sensitive patient data were handled safely and respectfully? Was there any potential for stigma or discrimination against participants arising from any of the procedures or outcomes of the study?*

All sensitive patient data were safeguarded by adhering to rigorous ethical standards and robust data protection protocols. Information that could identify participants was anonymised, and only authorised research staff could access it. The study underwent review by independent ethics committees to ensure that no procedures or dissemination activities would expose participants to risks of stigma or discrimination. Research personnel received training on local cultural considerations, and clear, informed consent was prioritised throughout the process. No individuals or communities were identified in a way that could result in harm, and dedicated feedback mechanisms were established to promptly address any concerns regarding privacy or confidentiality.

b) *Might any of the tests be experienced as invasive or culturally insensitive?*

Not applicable.

c) *How did you determine that work was sensitive to traditions, restrictions, and considerations of all cultural and religious groups in the study population?*

To ensure cultural and religious sensitivity, the study team consulted community leaders, health officials, and representatives from various groups to understand local customs and traditions. Protocols were then adapted based on their input. Ongoing feedback and staff training promoted respectful interactions, helping foster trust and inclusivity throughout the research.

d) *Were biowaste and radioactive waste disposed of in accordance with local laws?*

Yes.

e) *Were any structures built that would have impacted members of the community or the environment (such as handwashing facilities in a public space)? If so, how did you ensure that you had appropriate community buy-in?*

Not applicable.

f) *How might the study have impacted existing health-care resources (such as staff workloads, use of equipment that is typically employed elsewhere, or reallocation of public funds)?*

The study worked closely with local health authorities to minimise any strain on health-care resources. Dedicated research staff were used instead of drawing from clinical teams, and research equipment was sourced separately to avoid impacting regular patient care. Funding was obtained independently, so public health budgets were not affected. Any extra costs for health facilities due to the study were reimbursed as needed.

7. Confirm that local ethics review was sought, and please provide the approval number. If not sought, please explain why.

Bangladesh:

- National Research Ethics Committee, Bangladesh Medical Research Council (BMRC: 27406022020)
- Institutional Review Board, Johns Hopkins Bloomberg School of Public Health, Baltimore, MD, USA (11306)

Ethiopia:

- National Research Ethics Committee, Ministry of Education (RE/141/9148/21)
- Addis Ababa University, College of Health Sciences Institutional Review Board (050/20/SPH)

India, UP:

- Health Ministry's Screening Committee (HMSC), Department of Health Research (DHR), Indian Council of Medical Research (ICMR), New Delhi (2020-10119)
- Community Empowerment Lab (CEL) Institutional Ethics Committee (CEL/RES/202007/001)
- Ethics Committee, Ganesh Shankar Vidyarthi Memorial (GSVM) Medical College, Kanpur (CE/104/July/2020)
- Institutional Ethics Committee, Sarojini Naidu Medical College (SNMC), Agra (SNMC/IEC/2022/65)

India, HP:

- Health Ministry's Screening Committee (HMSC), Department of Health Research (DHR) of the Indian Council of Medical Research (ICMR), New Delhi (2021- 0047/F1)
- Ethics Review Committee, Society for Applied Studies, New Delhi for all research sites (SAS/ERC/PSBI-RCT-Study-1/2020)
- Institutional Ethics Committee, Dr YS Parmar, Government Medical College, Nahan, Himachal Pradesh (HFW/ME/DYSPGMC/IEC/2020/06)
- Institutional Ethics Committee, Indira Gandhi Medical College and Hospital, Shimla, Himachal Pradesh (HFW(MC-II) 13 (12)ETHICS/2020-15672)
- Biomedical Research Ethics Committee, Pt. BD Sharma PGIMS/UHS, Rohtak, Haryana (BREC/22/40)
- Institutional Ethics Committee, Dr Baba Saheb Ambedkar Medical College and Hospital, Delhi (5 (32)2020/BSAH)DNB/PF/22594-95)
- Institutional Ethics Committee, Lady Hardinge Medical College and Associated Hospitals, New Delhi (LHMC/IEC/2023/04)

Nigeria:

- Ahmadu Bello University Teaching Hospital Health Research Ethics Committee (ABUTHZ/HREC/W32/2020)

Pakistan:

- National Bioethics Committee (4-87/NBC-509/20/603)
- Institutional Review Board, The Aga University Ethics Review Committee (2020-3594-8920)

Tanzania:

- Institutional Review Board: National Institute Of Medical Research (NIMR/HQ/R.8a/Vol. IX/3492)
- Institutional Review Board: Muhimbili University of Health And Allied Sciences (MUHAS-REC-04-2020-081)
- Institutional Review Board, Tanzania Medicine and Medical Devices (TMDA0020/CTR/0001/05)
- Institutional Review Board, Harvard School of Public Health, Harvard University, Boston, MA, United States (IRB20-0119)

WHO:

- Ethics Review Committee for all research sites (ERC.0003289)

Secondary analyses

8. Have the data analysed in your study been extracted from another source, such as a national survey, rather than being directly collected by the authors of this paper?

No

If the authors of this paper were not involved in data collection, how were the findings interpreted with sufficient contextual knowledge?
The Lancet Global Health *believe contextual understanding is crucial for informed data analysis and interpretation.*

Not applicable.

9. Finally, please provide the title (eg, Dr/Prof, Mr/Mrs/Ms/Mx), name, and email address of an author who can be contacted about this statement.

Name: Dr Yasir Bin Nisar
Email: nisary@who.int
